# TWIST1 is a critical downstream target of the HGF/MET pathway and is required for MET driven acquired resistance in oncogene driven lung cancer

**DOI:** 10.1038/s41388-024-02987-5

**Published:** 2024-03-01

**Authors:** Vinod Kumar, Zachary A. Yochum, Princey Devadassan, Eric H.-B. Huang, Ethan Miller, Roja Baruwal, Purva H. Rumde, Autumn L. GaitherDavis, Laura P. Stabile, Timothy F. Burns

**Affiliations:** 1 Department of Medicine, Division of Hematology-Oncology, University of Pittsburgh School of Medicine, Pittsburgh, PA USA; 2 UPMC Hillman Cancer Center, Pittsburgh, PA USA; 3 Department of Pharmacology and Chemical Biology, University of Pittsburgh School of Medicine, Pittsburgh, PA USA; 4 Department of Medicine, Medical Oncology, Yale School of Medicine, New Haven, CT USA

**Keywords:** Non-small-cell lung cancer, Cancer therapeutic resistance

## Abstract

*MET* amplification/mutations are important targetable oncogenic drivers in NSCLC, however, acquired resistance is inevitable and the majority of patients with targetable *MET* alterations fail to respond to MET tyrosine kinase inhibitors (TKIs). Furthermore, *MET* amplification is among the most common mediators of TKI resistance. As such, novel therapies to target MET pathway and overcome MET TKI resistance are clearly needed. Here we show that the epithelial-mesenchymal transition (EMT) transcription factor, TWIST1 is a key downstream mediator of HGF/MET induced resistance through suppression of p27 and targeting TWIST1 can overcome resistance. We found that TWIST1 is overexpressed at the time of TKI resistance in multiple MET-dependent TKI acquired resistance PDX models. We have shown for the first time that MET directly stabilized the TWIST protein leading to TKI resistance and that TWIST1 was required for MET-driven lung tumorigenesis as well as could induce MET TKI resistance when overexpressed. TWIST1 mediated MET TKI resistance through suppression of p27 expression and genetic or pharmacologic inhibition of TWIST1 overcame TKI resistance in vitro and in vivo. Our findings suggest that targeting TWIST1 may be an effective therapeutic strategy to overcome resistance in MET-driven NSCLC as well as in other oncogene driven subtypes in which *MET* amplification is the resistance mechanism.

## Introduction

Lung cancer is the leading cause of cancer death among both men and women, and accounts for one third of all cancer deaths worldwide [1]. Recent advances in the treatment of non-small cell lung cancer (NSCLC) have come from recognition that NSCLC is not a single disease entity but rather a collection of distinct molecularly driven neoplasms. This has resulted in significant progress in the treatment of oncogene driven lung cancers such as *EGFR, BRAF, MET, KRAS and HER2* mutant and *ALK, ROS1, RET, NTRK1-3* translocation positive NSCLC with tyrosine kinase inhibitors (TKIs) targeting these oncogenes [2].

Approximately 4–6% of NSCLC patients harbor *MET* amplification or mutations which are important targetable oncogenic drivers [3]. The MET protein is a receptor tyrosine kinase which upon binding its ligand, hepatocyte growth factor (HGF), mediates tumor cell proliferation, epithelial-mesenchymal transition (EMT), motility, invasion, angiogenesis and metastasis [4]. The MET pathway is often dysregulated in NSCLC as a result of MET or HGF protein overexpression, *MET* genomic amplification or *MET* mutations [4]. *MET* amplification is found in 2–4% of primary NSCLC tumors [5, 6], and is a common mechanism of acquired resistance to epidermal growth factor receptor (EGFR) TKIs [7, 8] as well as other oncogenic drivers [9–11]. Despite the high frequency of MET amplification as the driver of acquired TKI resistance, the mechanism through which this pathway induces resistance has not been fully explored. In addition to *MET* amplification, *MET* mutations that result in exon 14 skipping have been identified in 2-4% of lung tumors [3, 5]. Both *MET* exon 14 skipping mutations and *MET* amplification are targetable alterations in NSCLC [12–15]. Despite the relatively high response rates of MET TKIs in *MET* exon 14 skipping mutant NSCLC (46-68%) and *MET* amplified NSCLC (40%) [12–16], acquired resistance is inevitable, a large percentage of patients with targetable *MET* aberrations fail to respond to MET TKIs, and a subset of *MET* alterations are untargetable with MET TKIs [17]. Multiple resistance mechanisms have been identified including on-target (MET-dependent), activation of bypass signaling pathways and histologic transformation including EMT or SCLC transformation [17–21]. The presence of a EMT or mesenchymal phenotype at the time of resistance [22, 23] suggests that mediators of EMT may contribute to resistance.

The EMT transcription factor (EMT-TF), TWIST1, is overexpressed in approximately 40% of NSCLC and is associated with a more aggressive tumor phenotype, increased risk of metastasis, and worsened patient prognosis [24]. We have previously demonstrated that TWIST1 expression is required for oncogene-driven NSCLC including many of the oncogenic drivers that lead to MET TKI resistance. Genetic and pharmacological inhibition of TWIST1 in NSCLC results in oncogene-induced senescence (OIS) and in a subset of cells, apoptosis [24–27]. Despite the importance of this transcription factor, it is unknown how TWIST1 expression is upregulated at the time of acquired resistance. In the current study, we explored the role of TWIST1 in mediating resistance to MET TKIs and EGFR TKIs and identified TWIST1 as a key downstream mediator of HGF/MET induced resistance through suppression of p27. We found that TWIST1 is overexpressed at the time of MET TKI resistance in a novel *MET* exon 14 mutant NSCLC patient derived xenograft (PDX) as well as in a second MET-dependent EGFR TKI acquired resistance PDX model [28, 29]. We then demonstrated that TWIST1 was required for tumor proliferation in *MET* altered NSCLC both in vitro and in vivo. Furthermore, we demonstrated that TWIST1 was required for HGF mediated tumorigenesis utilizing a novel genetically engineered mouse model (GEMM) which constitutively overexpress HGF and doxycycline inducibly express Twist1. In addition, we found that HGF induces TWIST1 expression by increasing protein stability in an ERK-dependent manner. As such, we have established that TWIST1 is a critical downstream target of the HGF/MET pathway as it was required both in vitro and in vivo for HGF/MET-driven lung cancer. We next demonstrated that sustained TWIST1 expression was sufficient to induce resistance to the FDA-approved MET TKIs (capmatinib, tepotinib and crizotinib) in vitro and in vivo in *MET* altered cell lines. TWIST1 expression led to suppression of MET TKI mediated p27 expression which is critical for MET TKI sensitivity as silencing of p27 was sufficient to induce resistance. Conversely, TWIST1 inhibition led to induction of p27 and overcame MET TKI resistance in vitro and in vivo. Finally, we demonstrated that targeting TWIST1 with our novel TWIST1 inhibitor, harmine in combination with erlotinib was sufficient to overcome MET-driven resistance in an *EGFR* mutant *MET* amplified PDX model. Our studies demonstrate that TWIST1 is a potential therapeutic target to overcome resistance to TKIs in MET driven NSCLC.

## Results

### TWIST1 was overexpressed in patient derived xenografts at the time of MET TKI resistance and can be targeted

**Fig. 1:**
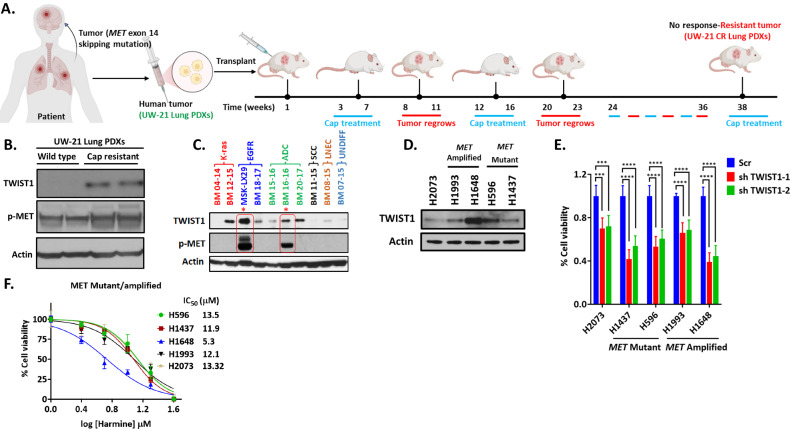
TWIST1 is overexpressed in patient derived xenografts (PDXs) at the time of MET TKI resistance and can be targeted. **A** Schematic diagram for the generation of capmatinib resistant PDXs in mouse model. The tumor from a patient harboring *MET* exon 14 mutant NSCLC brain metastasis (UW-21 lung) were directly implanted in mice and treated with capmatinib at an equivalent dose of 5 mg/kg animal body weight. The xenografts show complete response after 3-4 weeks of capmatinib treatment. The tumor subsequently regrew after 4–6 weeks. The mice were treated with 4-6 alternate cycle of capmatinib for three weeks each time until tumor was capmatinib resistance. The capmatinib resistant PDX was named UW-21 CR lung. The illustrations were created with BioRender.com. **B** Western blot demonstrating TWIST1 overexpression in capmatinib resistant PDXs (UW-21 CR lung) compared to their capmatinib sensitive tumor (UW-21 lung). **C** Western blot demonstrating that *MET* amplification leads to increased expression of TWIST1 in NSCLC PDXs. Encircled in red boxes are the PDXs with high p-MET. Red asterisk above the cell line lane denotes *MET* amplified cells. Higher TWIST1 expression in *MET* amplified PDXs resulted these PDXs resistance to MET TKIs (LNEC: large cell neuroendocrine carcinoma; ADC: adenocarcinoma; SCC: squamous cell carcinoma; UNDIFF: undifferentiated tumor). **D** Baseline expression of TWIST1 in *MET* wild type, *MET* amplified and *MET* mutated human cell lines. **E** Silencing of *TWIST1* with two distinct shRNAs resulted in growth inhibition in a *MET* mutant (H1437, H596) or *MET* amplified (H1993, H1648) cell lines as shown by cell viability assay (Scr, scrambled control for shRNA). **F** Cell viability assay demonstrating that the TWIST1 inhibitor, harmine has activity in a panel of *MET* altered NSCLC cells after 72 h of treatment.

To investigate if TWIST1 was induced at the time of MET TKIs resistance, we first generated a novel capmatinib resistant PDX from a previously characterized PDX (UW-21-lung) [28] from a patient with a *MET* exon 14 mutant NSCLC brain metastasis. Previously, this tumor was demonstrated to be sensitive to the MET inhibitor savolitinib alone or in combination with radiation [28]. We therefore, tested the therapeutic response of UW-21 lung PDXs with FDA approved MET TKI inhibitor capmatinib. We treated the tumor for 3–4 weeks with capmatinib at an equivalent dose of 5 mg/kg animal body weight. The tumor responded completely and disappeared. The mice was kept under observation and tumors regrew after 4–6 weeks. At time of regrowth, it was again treated with capmatinib for three weeks until all the tumor disappeared. This cycle was repeated at regrowth for 4–6 times until we observed that tumors were no longer responsive to capmatinib treatment. The resulting capmatinib resistant PDX was named UW-21 CR lung (Fig. 1A). Our western blot data show that both capmatinib sensitive (UW-21 lung) and capmatinib resistant tumors (UW-21 CR lung) had positive staining for p-MET and the TWIST1 expression was significantly higher in capmatinib resistant PDXs (Fig. 1B). These data show that TWIST1 is overexpressed at the time of MET TKI resistance. We next examined if TWIST1 expression was higher in a variety NSCLC patient derived xenografts derived from both primary tumors (MSK-LX29) [29] and brain metastases from *KRAS mutant* [30] or *EGFR mutant* adenocarcinomas, squamous cell carcinoma, large cell neuroendocrine carcinoma, and undifferentiated NSCLC tumors. We found TWIST1 expression in majority of these PDXs (Fig. 1C). Interestingly, 2 of the 4 high expressing PDXs had *MET* amplification (Supplementary Fig. 1A) [29] and high levels of pMET (MSK-LX29 and BM16-16). MSK-LX29 tumor [29] was derived from the patient with *EGFR* mutant NSCLC who developed resistance after 3 months of erlotinib treatment with an acquired *MET* amplification and had the highest TWIST1 expression (Fig. 1C). All of the above data support that TWIST expression is associated with acquired resistance to TKIs.

We next examined TWIST expression in a panel of *MET* mutant/amplified human cell lines (Fig. 1D). We included both *MET* mutant (H1437 and H596) and *MET* amplified (H1993 and H1648) cell lines. Of note, a subset of the cell lines (H596 and H1437) which are widely used in the field [21, 31–36], have limited response to MET TKIs [21, 36, 37] with IC50s in micromolar range (Supplementary Fig. 2). The limited sensitivity of H596 cells in part is likely due to presence of a PIK3CA mutation [21, 36, 37]. Furthermore, as the response rate of FDA approved MET TKIs (capmatinib, tepotinib) in the treatment naïve setting is only 50–60%, these less sensitive *MET* altered cell lines are important to include in these studies [12, 14] and can consider models of de novo resistance. Interestingly, we found that TWIST1 levels were higher in less MET TKI sensitive cell lines (H596 and H1648) (Fig. 1D). Our group has previously demonstrated that TWIST1 is required for oncogene-driven NSCLC including *KRAS* and *EGFR* mutant NSCLC [25–27]. Since TWIST1 is expressed at the time of TKI resistance and in MET altered cell lines, we next examined whether TWIST1 was required in *MET* altered human cell lines. Similar to our prior findings, we demonstrated that genetic inhibition of TWIST1 inhibited growth in *MET* amplified and *MET* mutant NSCLC cell lines (Fig. 1E and Supplementary Fig. 1). We previously identified and characterized a novel TWIST1 inhibitor, harmine, that degrades TWIST1, inhibits multiple TWIST1-dependent functions, and results in growth inhibition in oncogene-driven NSCLC [26, 27]. Similar to genetic loss of TWIST1, harmine treatment results in growth inhibition in *MET* amplified and *MET* mutant NSCLC cells (Fig. 1F). Interestingly, *MET* amplified/mutant NSCLC cell lines with higher basal levels of TWIST1 expression such as H1648 appear to be more sensitive to TWIST1 inhibition (Fig. 1F).

### TWIST1 is required for HGF-MET-driven NSCLC tumorigenesis

**Fig. 2:**
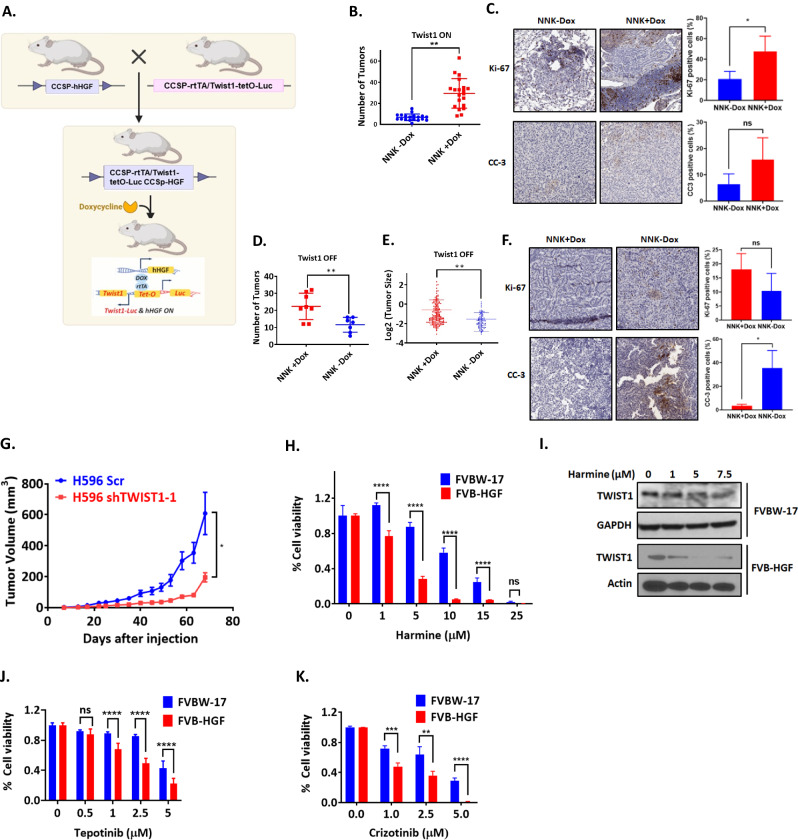
TWIST1 is required for HGF-MET driven NSCLC in vivo. **A** Schema representing the generation of genetically engineered mouse model ***C****CSP-rtTA*/ ***T****wist1-tetO-luc /CCSP*-***H****gf* (**CTH**) by crossing ***C****CSP-****H****gf* (**CH**) mice with ***C****CSP-rtTA/****T***wist1-tetO-luc (**CT**) mice. CTH mice constitutively overexpress HGF and doxycycline inducibly express Twist1. TWIST1 expression leads to increased tumor number (**B**) in a NNK induced ***C****CSP-rtTA/* ***T****wist1-tetO-luc /CCSP-****H****gf* (**CTH**) lung cancer model. In this cohort, at 6 weeks of age, half of the mice were placed on doxycycline containing chow and half of the mice were given regular chow till the end of experiment. After two weeks, both group of mice received NNK twice per week (3 mg per i.p. injection) for 5 weeks (6 mg/week). Following NNK treatment, mice were observed for 17 weeks and then harvested for tumor analysis. **C** Representative images of Ki-67 and cleaved caspase-3 (CC-3) IHC staining and quantification (right) of staining showing increase in proliferation and apoptosis in mice treated with doxycycline. In a separate cohort (**D**, **E**), all mice received doxycycline and NNK at the indicated doses above and then at 17 weeks after NNK, half of the mice were randomized to continue doxycycline (Twist1-ON) and half were placed on regular chow (Twist1 OFF) for four weeks at which point the lungs were harvested. Lungs were also harvested at two weeks after doxycycline withdrawal in a subset of animals that were used for the indicated studies in (**E**) and (**F**). Withdrawal of Doxycycline (Twist1 OFF) for four weeks (**D**, **E**) or two weeks (**F**) leads to decrease in the tumor number (**D**) and size (**E**), decrease in proliferation (ns, *p* = 0.1878) and increase in apoptosis (*p < 0.05) by IHC (**F**). **G** Silencing of *TWIST1* with shRNA resulted in significant tumor growth inhibition in xenograft mouse model of *MET* exon 14 skipping mutant cell line H596. Data represent mean ± SEM. *p < 0.05, **p < 0.01. 2-tailed Student’s t-test. **H** HGF overexpressing cells derived from genetically engineered mouse model (GEMM) are more sensitive to TWIST1 inhibition as demonstrated by harmine treatment for 48 h in FVBW-17 and FVB-HGF cells in cell viability assays. **I** Western blot demonstrating decrease TWIST1 levels after harmine treatment in FVBW-17 and FVB-HGF cells. FVB-HGF cells display increased sensitivity to MET inhibition (tepotinib (**J**) and crizotinib (**K**) compared to FVBW-17 cells in cell viability assays. Data represent mean ± SD (n = 4 technical replicates). *P < 0.05, **P < 0.01, ***P < 0.001,****P < 0.0001, two-way ANOVA.

Since TWIST1 is overexpressed the time of TKI resistance and is required for *MET* altered NSCLC in vitro, we next evaluated the dependency of MET driven NSCLC on TWIST1 in vivo. To study this, we created a novel HGF-TWIST1 overexpressing genetically engineered mouse model (GEMM). Previously, we demonstrated that HGF overexpression in the lung epithelium enhanced carcinogenesis induced by exposure to nitrosamine 4-(methylnitrosamino)-1-(3-pyridyl)-1-butanone (NNK) [38] as these mice (CCSP-hHGF) developed more tumors when compared to wild-type mice [38]. In addition, the resulting tumors were MET-dependent as they were sensitive to MET inhibition [38]. In the current study, we crossed our CCSP-hHGF mice with our CCSP-rtTA/Twist1-tetO-Luc, which doxycycline-inducible overexpresses murine Twist1 in the lung epithelium [24, 26]. The resulting **C**CSP-rtTA/**T**wist1-tetO-Luc/CCSP-h**H**GF mice (**CTH**) constitutively overexpress HGF and doxycycline-inducibly overexpress TWIST1 in the lung epithelium (Fig. 2A). Using the CTH model, we demonstrated that doxycycline treatment (Twist1 ON) leads to an increased number of tumors (a mean of 6.7 tumors/mice was observed in NNK-Dox group versus a mean of 29.5 tumors/mice observed in NNK+Dox group) after NNK exposure (Fig. 2B). A significant increase in the number of tumors was observed in both males and females but no statistically significant sex difference were observed in the relative increase in tumors (interaction term *p* = 0.13) (Supplementary Fig. 3). Furthermore, these tumors showed a statistically significant increase in proliferation (Ki-67) but not apoptosis (CC-3) (Fig. 2C). We next asked whether these tumors were Twist1-dependent. To test this, we removed doxycycline for 4 weeks in a cohort of mice (Twist1 OFF) and demonstrated a significant decrease in the number of tumors (93% reduction in tumor number (95% confidence interval 49–151%) Poisson regression, *p* < 0.001) (Fig. 2D) and size of tumors (a 58% reduction in tumor size (95% confidence interval 18–112%) (linear mixed effects regression, p = 0.002) (Fig. 2E) after the doxycycline was withdrawn. We failed to see a significant change in proliferation, however, we observed a significant increase in apoptosis in the Twist1 OFF tumors (Fig. 2F). To further explore the role of TWIST1 in MET driven NSCLC in vivo, we silenced TWIST1 in the *MET exon 14 skipping* mutant NSCLC cell line H596 and found that genetic inhibition of TWIST1 led to a significant decrease in tumor growth (68% reduction in mean tumor size) (Fig. 2G). Overall, these data demonstrate that TWIST1 is required for HGF-MET driven NSCLC tumorigenesis in vivo.

We next, derived cell lines from the resulting tumors in these transgenic animals and examine whether they were Twist1 dependent. Remarkably, the FVB-HGF cells derived from tumors from our lung specific Hgf expression transgenic were significantly more sensitive to our TWIST1 inhibitor, harmine compared to our cell line derived from wild-type mice (FVBW-17 cells) [39] (Fig. 2H). This increased sensitivity to harmine in FVB-HGF cells correlated with increased baseline TWIST1 expression and greater TWIST1 degradation after harmine treatment (Fig. 2I). FVB-HGF cells were also more sensitive to *MET* inhibitor treatment suggesting that these cells are MET-dependent (Fig. 2J, K). These observations demonstrate that MET-driven lung cancer cell lines are dependent on TWIST1 and that TWIST1 is a potential therapeutic target in MET-driven NSCLC.

### HGF induces TWIST1 expression via a post-translational mechanism

**Fig. 3:**
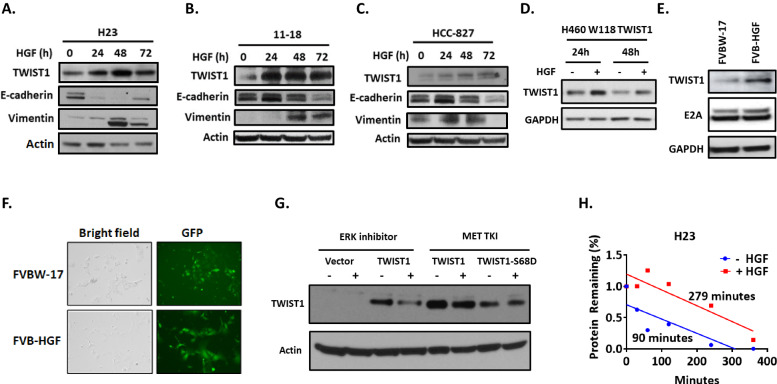
HGF induces TWIST1 expression and EMT in NSCLC. **A** Induction of endogenous TWIST1 and additional EMT markers after HGF treatment in *MET* wild type Non-Squamous NSCLC cell lines (**A**) H23 (**B**) 11-18 (**C**) HCC-827 or exogenously expressed TWIST1 in **D** H460 cells. **E** HGF overexpression drive TWIST1 and E2A expression in murine NSCLC cells derived from carcinogen induce lung tumors in wild type FVB/N (FVBW-17) or transgenic mouse model with lung specific Hgf expression (FVB-HGF) respectively. **F** Microscopic images (20X) of FVBW-17 and FVB-HGF cells, phase-contrast (left) and fluorescence microscopy (right). The FVB-HGF cells have a more mesenchymal phenotype compared to FVBW-17 cells. **G** HGF treatment stabilizes TWIST1 through an ERK-dependent manner. H1993 cells overexpressing either wild type or S68D TWIST1 protein were treated with either an ERK inhibitor (LY3214996, 250 nM) or MET inhibitor (Capmatinib, 50 nM) and protein was collected at 48 h and western blot performed. **H** HGF regulates TWIST1 expression at the posttranslational level. HGF treatment increases the half-life of TWIST1 protein in H23 cell line. Protein concentration was quantified using densitometry and protein half-lives were determined using linear regression.

HGF is frequently overexpressed both in MET driven and *EGFR* mutant NSCLC at the time of TKI resistance [40, 41] and HGF expression correlates with a poor prognosis [42]. HGF plays a critical role in cell motility, cell growth and angiogenesis and can induce EMT in NSCLC [43, 44]. As we have shown that the EMT-TF, TWIST1 is highly expressed in *MET* mutant/amplified cell lines (Fig. 1D), is required for survival in *MET* altered cell lines, and can induce EGFR TKI resistance to a similar extent as HGF treatment [27], we decided to explore whether HGF could induce TWIST1 expression. To study the effect of HGF on the protein level of TWIST1, we first treated non-*MET* altered human NSCLC cell lines with HGF. We demonstrated that HGF treatment increases the expression of endogenous TWIST1 in NSCLC cell lines with *KRAS* and *EGFR* mutations (Fig. 3A–C). Along with increased TWIST1 expression, exogenous HGF treatment resulted in increased expression of mesenchymal marker Vimentin and decreased expression in epithelial marker E-Cadherin (Fig. 3A–C). Using a NSCLC cell lines that overexpresses TWIST1, we found that HGF treatment also increases expression of exogenous TWIST1 (Fig. 3D). As we had previously shown that the TWIST1 dimer partner, E2A is critical for TWIST1 function and stability [26], we next asked whether HGF lead to increased E2A levels. We observed increased basal expression of TWIST1 and E2A in FVB-HGF cells compared to wild type cells (FVBW-17 cells) (Fig. 3E). Interestingly, FVB-HGF cells also adopt a more mesenchymal phenotype when compared to the wild-type control cells (Fig. 3F).

We next investigated the mechanism(s) by which HGF regulates TWIST1 expression. Previous studies have demonstrated the MAPK-ERK pathway increases TWIST1 protein stability [45]. Here, we demonstrated that pharmacologic inhibition of either the ERK and MET pathways decrease TWIST1 protein expression (Fig. 3G). Interestingly, MET inhibition does not result in a decrease in expression of a phospho-mimetic Serine 68 (S68D) TWIST1 mutant (Fig. 3G), which has been previously demonstrated to be an ERK phosphorylation site (Supplementary Fig. 4A) [45]. We found that HGF treatment does not result in an increase in *TWIST1* mRNA expression (Supplementary Fig. 4B) but did increase the half-life of the TWIST1 protein suggesting that HGF regulates TWIST1 via a post-translational mechanism (Fig. 3H). Altogether, this suggests that the HGF-MET pathway increases TWIST1 expression likely through an ERK-dependent phosphorylation of TWIST1 leading to increased protein stability.

### TWIST1 induces resistance to MET TKIs in MET-driven NSCLC and prevents MET TKI-dependent p27 induction

**Fig. 4:**
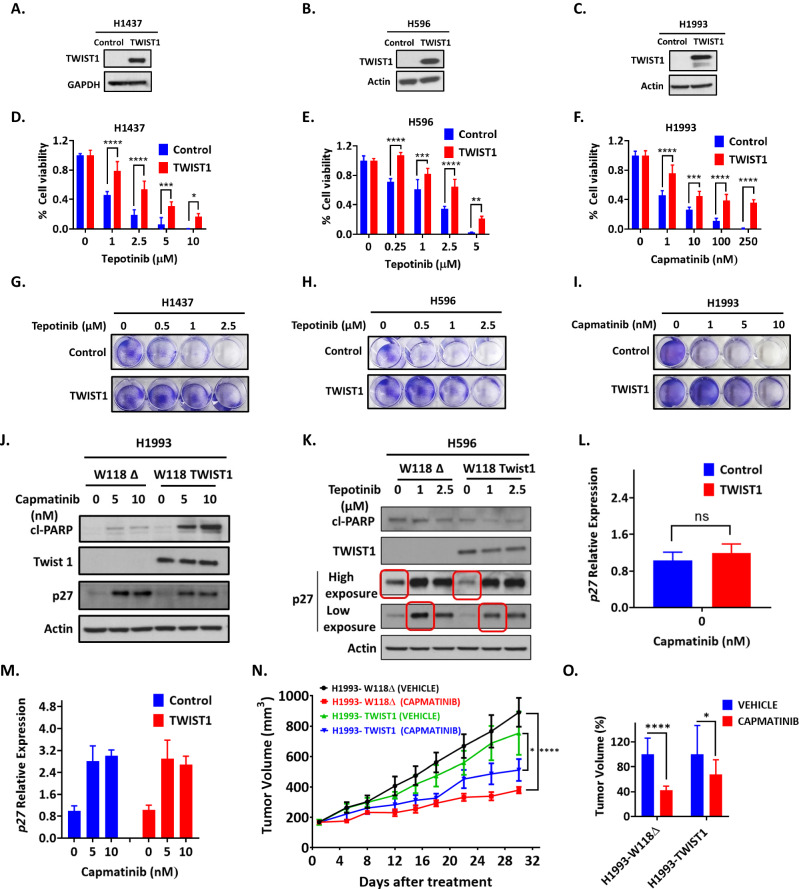
TWIST1 over expression induces MET TKI resistance. Stable cell lines were made by infecting lentivirus encoding for Twist1 and control vector and selecting the cells with puromycin (**A**) H1437 (**B**) H596 (**C**) H1993. **D**–**I** Cell-Titer Glo and colony formation assay demonstrating that TWIST1 overexpression decreases response to TKIs in **D**, **G** H1437, **E**, **H** H596, and **F**, **I** H1993 cell lines. Data represent mean ± SD (n = 4 technical replicates). *P < 0.02, **P < 0.001, ***P < 0.0002,****P < 0.0001, two-way ANOVA. The cells were treated with the indicated doses of TKIs and stained after 15 days following treatment. (In colony formation assay, for full triplicate plate picture reader is referred to Supplementary Fig. 5, only cropped version is presented here). Western blot analysis demonstrating decreased MET TKI induction of p27 in TWIST1 overexpressing **J** H1993 and **K** H596 cell lines after MET TKI treatment. Encircled in red boxes are the panel of p27 expression which has maximum fold change (W118Δ vs W118 TWIST1). Real time PCR data of *p27* expression in TWIST1 overexpressed H1993 cells. *p27* mRNA expression was measured in absence (**L**) and presence (**M**) of capmatinib. TWIST1 expression does not changes *p27* mRNA expression level in absence of capmatinib treatment. Data represent mean ± SD (n = 3 technical replicates). paired t-test. ns=non significant. **N** TWIST1 overexpression in vivo is sufficient to cause capmatinib resistance in a H1993 xenograft model. TWIST1 overexpressing (H1993-W118 TWIST1) stable cell lines and the control cell lines (H1993-W118Δ) were established. One million each cell was injected subcutaneously. Capmatinib was administered by oral gavage five times a week for 4 weeks at an equivalent drugs dose of 5 mg/kg animal body weight. **O** TWIST1 overexpression decreased tumor regression after capmatinib treatment compared to empty vector control. Each group tumor was normalized with their respective control. ****P < 0.0001, two-way ANOVA.

We have previously shown that TWIST1 can induce EGFR TKI resistance in vitro and in vivo through suppression of apoptosis [27]. In contrast, the role of EMT-TFs in *MET* TKI resistance is poorly understood. We have found that inhibition of TWIST1 was sufficient to inhibit oncogene-driven lung tumorigenesis [24–26] and in this current study, MET-driven NSCLC (Figs. 2 and 3). Since HGF can induce TWIST1 and TWIST1 is required in HGF-MET-driven NSCLC, we next asked if sustained TWIST1 expression in the presence of MET TKI would be sufficient to induce *MET* TKI resistance in NSCLC. To test this, we first generated doxycycline inducible TWIST1 over expressing *MET* amplified H1993 cells. Even though TWIST1 was overexpressed in presence of doxycycline at baseline, we did not observe any difference in cell viability after capmatinib treatment (Supplementary Fig. 5A) and p27 was induced to similar levels after MET TKI treatment (Supplementary Fig. 5B). This was likely due to the presence of a weak promoter in the doxycycline inducible vector system as MET TKI treatment still led to TWIST1 degradation in the presence of doxycycline. Therefore, we next established stable TWIST1 overexpressing cell lines by using lentivirus-based transduction in *MET* altered NSCLC cell lines with expression of the transgene under a CMV promoter. (Fig. 4A–C). Similar to others finding [21, 36, 37], we also reported that the cell lines H596 and H1437 are less sensitive to MET TKIs and have IC50s in micromolar range (Supplementary Fig. 2). In short-term proliferation assay experiments, we now found that sustained TWIST1 expression further decreased sensitivity against the MET TKIs capmatinib, crizotinib and tepotinib in both less sensitive (H596 and H1437) and sensitive (H1993) cell lines above their baseline level compared to their respective control cell lines (Fig. 4D–F, and Supplementary Fig. 5C–H). To evaluate the long-term effect of MET TKIs in these TWIST1 overexpressing cells, we performed clonogenic assay in which vector control or TWIST1 overexpressing cells were cultured in presence of MET TKIs for 15 days. Our results suggest that sustained TWIST1 expression substantially enhanced long-term survival in the presence of MET TKIs (Fig. 4G–I and Supplementary Fig. 5J–L). To explore the mechanism by which TWIST1 expression causes MET TKI resistance in *MET* altered cell lines, we next examined cell cycle marker (p27) and apoptotic protein (cleaved PARP) expression in these cells. We found that sustained TWIST1 expression caused suppression of p27 protein in both the presence and absence of MET TKIs in *MET* altered H1993, H596 and H1437 cell lines compared to their respective control cell lines (Fig. 4J, K and Supplementary Fig. 5I). This appears to be through a post-translational mechanism as TWIST1 overexpression did not decrease p27 (*CDKN1B)* mRNA expression nor inhibited capmatinib induced p27 mRNA expression (Fig. 4L, M). There was no significant change observed for cleaved PARP expression except in H1993 cells. In H1993-TWIST1 cells, increased cleaved PARP expression was seen with capmatinib, however, the cells were still less responsive to capmatinib (Fig. 4J). Taken together, our findings revealed that sustained expression of TWIST1 was sufficient to decrease MET TKI sensitivity in both less sensitive (H596 and H1437) and sensitive cell lines (H1993). These results suggest that TWIST1 can mediate de novo resistance in our MET TKI sensitive cell line (H1993) as well as in our less MET TKI sensitive cell lines.

We next examined whether sustained TWIST1 expression affected sensitivity to capmatinib in vivo. To test this, we implanted H1993-W118Δ and H1993 TWIST1 cells subcutaneously into the hind flank of NOD/SCID animals. Control H1993 (H1993-W118Δ) tumors were sensitive to capmatinib as evidenced by a 57.4% decrease in tumor volume after drug treatment (Fig. 4N, O). On the contrary, H1993-TWIST1- tumors were much less sensitive to capmatinib with only a 32.1% decrease in tumor volume (Fig. 4N, O). These results suggest that capmatinib inhibited tumor growth less effectively in H1993-TWIST1 than in H1993-W118 Δ tumors. Collectively, these findings strongly revealed that the sustained TWIST1 is sufficient to cause resistance to MET TKIs both in vitro and in vivo.

### Suppression of p27 is required for TWIST1 induced MET TKI resistance in *MET* altered NSCLC

**Fig. 5:**
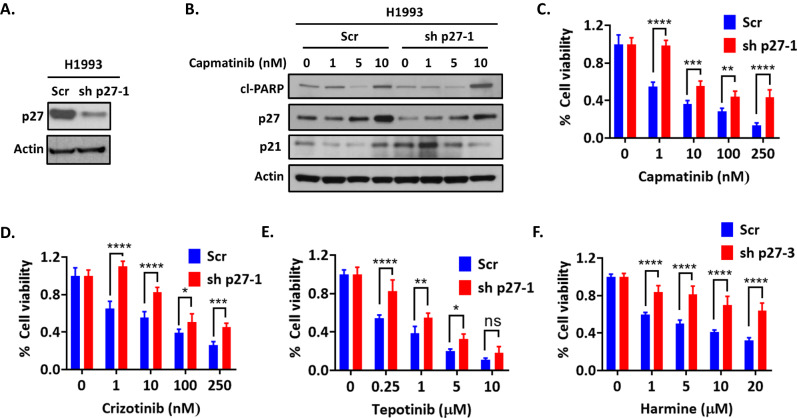
p27 is required for MET TKI and harmine induced cytotoxicity. **A** Western blot demonstrating partial knockdown of p27. **B** Western blot demonstrating that capmatinib treatment induces less p27 in shp27 H1993 cells compared to control (Scr) cells. Cell-Titer Glo assay demonstrating that p27 knockdown decreases response to **C** capmatinib **D** crizotinib **E** tepotinib. and **F** harmine Data represent mean ± SD (n = 4 technical replicates). *P < 0.05, **P < 0.01, ***P < 0.001,****P < 0.0001, two-way ANOVA.

To investigate the underlying mechanism how sustained TWIST1 expression causes MET TKI resistance, we next generated stable p27 knockdown H1993 cells since p27 expression was diminished both in vitro and in vivo with sustained TWIST1 expression (Fig. 4J–M and Supplementary Fig. 5I). Since p27 serves not only a critical role in cell cycle arrest through inhibition of cyclin dependent kinase (CDK) but also a role as an assembly factor for CDK-Cyclin complexes for cell cycle progression, p27 expression is rarely fully lost in cancer and complete knockdown of p27 is challenging [46]. Lentiviral based transduction of shp27 RNAs generated a partial knockdown p27 stable H1993 cell line (Fig. 5A and Supplementary Fig. 6A) which had diminished levels of p27 after capmatinib treatment compared to scrambled (scr) control cells (Fig. 5B). Interestingly, higher levels of p21 were found in p27 knockdown cells at baseline and after capmatinib treatment compared to control cells, suggesting the existence of a potential compensatory mechanism (Fig. 5B). To determine whether p27 was required for capmatinib sensitivity, we examined the sensitivity of these cells to MET TKIs in both short-term and long-term proliferation assays. We observed that shp27 cells were resistance to MET TKIs compared to scrambled control (Fig. 5C–E and Supplementary Fig. 6B–D) in short-term assays and had a substantially enhanced survival after capmatinib compared to control cells in long-term colony formation assays (Supplementary Fig. 6F). Interestingly, these shp27 cells were also resistance to TWIST1 inhibition (harmine) as well (Fig. 5F and Supplementary Fig. 6E). These data indicated that suppression of p27 is required for TWIST1 induced MET TKI resistance.

### The p27 protein is decreased at the time of acquired resistance to MET TKIs

**Fig. 6:**
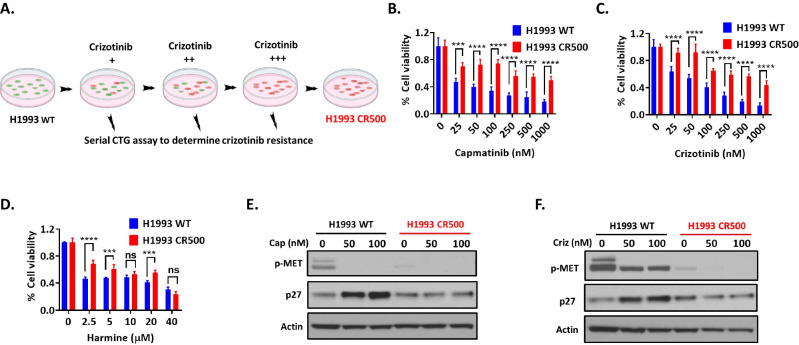
Acquired resistance to MET TKIs leads to suppression of p27 in *MET* amplified H1993 NSCLC cell lines. **A** Schematic diagram for the generation of acquired crizotinib-resistant H1993 (H1993 CR500) NSCLC cells. The H1993 CR500 cells were generated by exposing the corresponding wild-type (WT) cells to an increasing concentration of crizotinib over 6 months. The resistance status was confirmed by performing CTG assays at each step. Green and red colors of cells in the illustration denote sensitivity (or cell death) and resistance (or cell survival), respectively, based on the response to crizotinib treatment for 72 hr. Relative sensitivity of H1993 WT cells were compared with the H1993 CR500 cells as determined by CTG cytotoxic assay against **B** capmatinib **C** crizotinib and **D** harmine. Cells were treated with increasing concentration of capmatinib, crizotinib, harmine and CTG assays were performed 72 h after treatment. Representative western blot of H1993 WT and H1993 CR500 cell lysates treated with increasing concentrations of **E** capmatinib or **F** crizotinib suggest that MET TKI acquired resistance in H1993 cells is mediated by a suppression of p27 protein.

To investigate effect of TWIST1 inhibition and mechanistic basis of TKI resistance, we generated *MET* amplified human cell line H1993 with acquired crizotinib resistance. For this, *MET* amplified H1993 cells were cultured with increasing concentration of crizotinib over a period of approximately 6 months (Fig. 6A). The resistance status at each dose was determined by CTG cell cytotoxic assay (Fig. 6B, C). At the end of 6 month treatment period, the cell lines generated (H1993 CR500) were approximately 500 to 1000 fold more resistant compared with WT cells. Primarily, the H1993 acquired resistance cells were generated against crizotinib but we found that the cells were resistant to all three FDA approve *MET* TKIs capmatinib, crizotinib and tepotinib (Fig. 6B, C). We observed that acquired resistant H1993 cells remained sensitive to TWIST1 inhibitor harmine (Fig. 6D). Since higher TWIST1 expression downregulates p27 protein (Fig. 4J, K), we next measured the p27 expression in H1993 CR500 cells. Our western blot data show that the p27 protein expression was significantly diminished in resistant cell lines in presence of capmatinib and crizotinib when compared to wild type H1993 cells. These data support our findings of TWIST1 causing MET TKI resistance via suppression of p27 and p27 is essential for TWIST1 mediated MET TKIs resistance (Figs. 4 and 5). Taken together, these data suggest that suppression of p27 expression is essential for TWIST1 mediated MET TKI resistance both in de novo and acquired resistance condition.

### Targeting TWIST1 increases MET-driven NSCLC sensitivity to MET TKIs and overcomes resistance

**Fig. 7:**
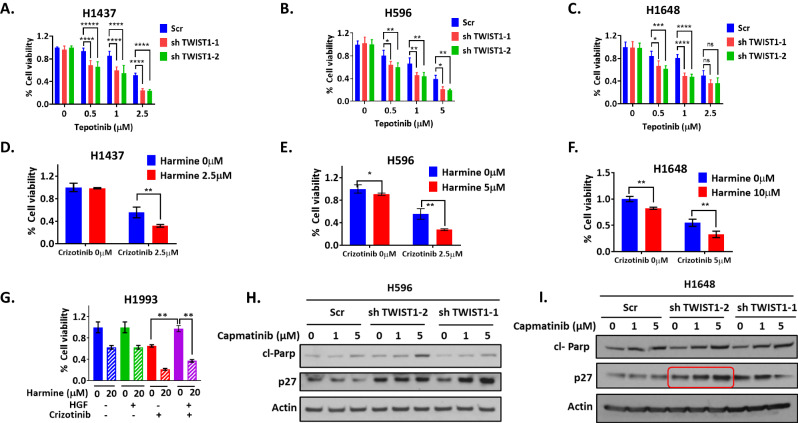
TWIST1 inhibition sensitizes to MET TKIs and overcomes MET TKI resistance in *MET* altered NSCLC. **A-F** Cell viability assay demonstrating that genetic and pharmacological inhibition of Twist1 with shRNA and harmine overcomes resistance to tepotinib and crizotinib in H1437 (**A, D**), H596 (**B**, **E**) and H1648 (**C**, **F**) cell lines respectively. **G** Representative cell viability assay demonstrating that in *MET* amplified H1993 cells harmine overcomes HGF-mediated resistance to MET targeted therapy. Cells were co-treated with the indicated doses of harmine, HGF (50 ng/ml), crizotinib (100 nM) for 72 h, and then harvested for MTS analysis. Data represent mean ± SD (n = 4 technical replicates). *P < 0.05, **P < 0.01, ***P < 0.001,**** P < 0.0001, two-way ANOVA. Western blot demonstrating induction of p27 after Twist1 inhibition with shRNAs followed by capmatinib treatment in H596 (**H**) and **I** H1648 cell lines respectively. Encircled in red boxes are the panel of p27 expression which has maximum fold change (Scr vs shTWIST1-2).

To further validate the role of TWIST1 in MET TKI resistance, we examined whether the TWIST1 inhibition could restore sensitivity to MET TKIs. We used lentivirus based TWIST1 shRNAs (shTWIST1-3) [24–27] and harmine respectively to inhibit TWIST1 both genetically and pharmacologically in *MET* altered cell lines (H1437, H596 and H1648). Cell viability analysis showed that TWIST1 inhibition enhanced sensitivity of MET TKIs both in MET TKI less sensitive (H596, H1437, H1648) and sensitive (H1993) (Fig. 7G) cell lines (Fig. 7A–F and Supplementary Fig. 7A–F). Moreover, HGF induced MET TKI resistance was abrogated by pharmacological inhibition of TWIST1, evidenced by resensitization of H1993 cells to crizotinib when treated with harmine (Fig. 7G). These results indicated that TWIST1 inhibition could sensitize *MET* altered cell lines to MET TKIs and overcome HGF induced MET TKI resistance.

We next examined the effect of TWIST1 inhibition on p27 protein expression in presence or absence of MET TKIs. Our western blot analysis demonstrated an upregulation of p27 protein at baseline after silencing of TWIST1 (Fig. 7H, I). Furthermore, significant upregulation of p27 was observed compared to scrambled control cells after capmatinib treatment (Fig. 7H, I). Similar results were observed in the H1437 cell line (Supplementary Fig. 7G). Collectively, these findings demonstrate that TWIST1 inhibition could resensitize cells to MET TKIs by upregulating p27 expression in MET driven NSCLC.

### TWIST1 inhibition increases MET-driven NSCLC sensitivity to TKIs in an EGFR TKI resistant *EGFR* mutant *MET* amplified patient derived xenograft

**Fig. 8:**
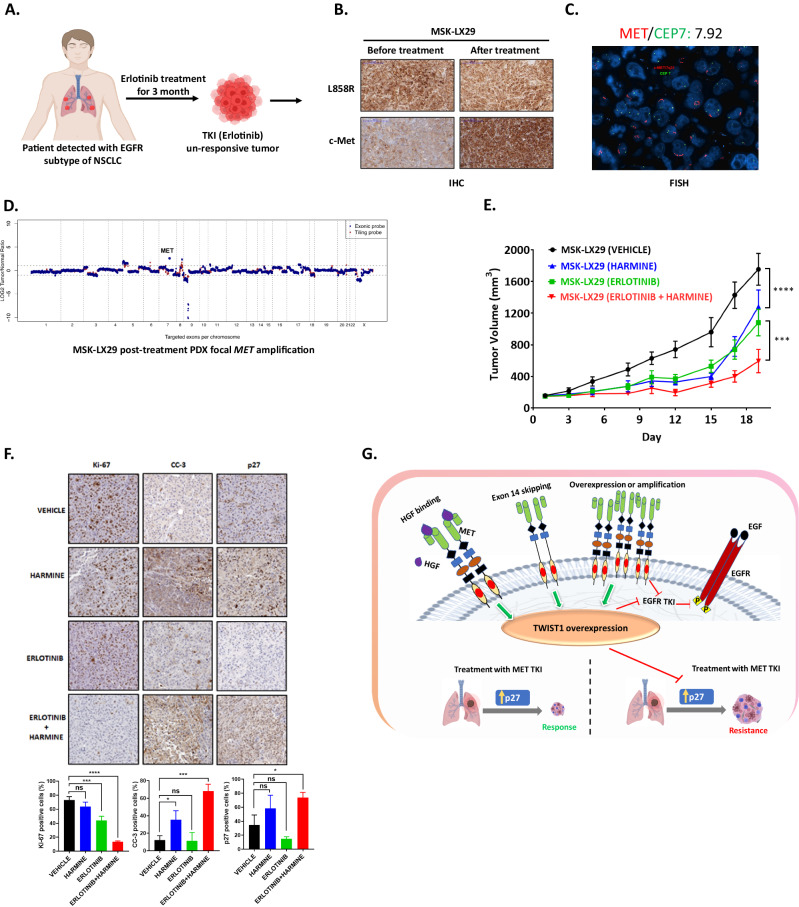
Harmine treatment resensitizes an EGFR TKI resistance *EGFR* mutant *MET* amplified tumor to erlotinib. **A** Generation and characterization of *MET* amplified, erlotinib (EGFR TKI) resistance, *EGFR*^*L858R*^ mutated patient derived xenograft (MSK-LX29) from a tumor biopsy from a patient with *EGFR* mutant NSCLC who developed resistance after 3 months of erlotinib treatment secondary to an acquired *MET* amplification [29]. The illustrations were created with BioRender.com. Tumor was characterized by **B** immunohistochemistry (IHC), **C** fluorescent in situ hybridization (FISH), and **D** *MET* copy number analysis. **E** Harmine overcomes resistance to EGFR TKI therapy in a PDX model (MSK-LX29) derived from a *EGFR*^*L858R*^ mutated *MET* amplified tumor [29]. Harmine was administered intraperitoneally at a doses of 10 mg/kg and erlotinib was administered by oral gavage at a dose of 25 mg/kg. Alone PBS was injected in control group. Data represent mean ± SEM. *P < 0.05, **P < 0.01, ***P < 0.001,**** P < 0.0001, 2-tailed Student’s t-test. **F** IHC staining (top) and quantification (bottom) showing decrease in proliferation level as measured by Ki-67 staining and increase in apoptosis (CC-3) and p27 levels in mice treated with MET TKI+ harmine compared to control group. Data represent mean ± SD (n = 3 technical replicates). *P < 0.05, **P < 0.01, ***P < 0.001,****P < 0.0001, 2-tailed Student’s t-test. **G** Model of TWIST1 mediated MET TKI resistance through suppression of p27 in NSCLC. MET activation is initiated through binding its ligand, HGF. In addition, *MET* amplification, mutation leads to higher level of MET expression and typically self-dimerize in a ligand-independent manner. MET dysregulation result into TWIST1 overexpression and sustained TWIST1 expression after MET TKIs causes resistance to MET TKIs via suppression of p27. *MET* amplification is also a common mechanism of acquired resistance to EGFR TKIs in *EGFR* mutant NSCLC.

We next evaluated whether the pharmacological inhibition of TWIST1 could overcome TKI resistance in a PDX model of acquired resistance. We assessed the therapeutic efficacy of our TWIST1 inhibitor harmine alone or in combination with erlotinib (EGFR TKI) in a *EGFR*^*L858R*^ mutant and acquired *MET* amplification PDX model, MSK-LX29 [29]. The tumor was derived from a patient who received erlotinib for 3 months and then developed acquired resistance secondary to an acquired *MET* amplification (Fig. 8A). Our IHC data confirmed the presence of *EGFR*^*L858R*^ mutation both before and after erlotinib treatment and higher MET expression at the time of resistance (Fig. 8B). We further confirmed *MET* amplification using FISH (*MET/CEP7* ratio= 7.92) and exome sequencing (Fig. 8C, D). Levels of pMET and TWIST1 were also increased in this PDX (Fig. 1C). Since MSK-LX29 was resistant to single agent EGFR and MET TKIs crizotinib but was sensitive to the combination [29], we therefore assessed the therapeutic efficacy of TWIST1 alone and in combination with erlotinib in vivo. Consistent with our in vitro results, treatment with harmine or erlotinib alone modestly reduced tumor growth (26.7% growth inhibition in harmine group and 38.3% growth inhibition in erlotinib group); however combined treatment with erlotinib plus harmine significantly inhibited tumor growth (66.1% growth inhibition) (Fig. 7E). The combination treatment significantly inhibited tumor cell proliferation marked by a significant decrease in Ki-67 staining compared to monotherapy. CC-3 was significant increased after harmine treatment and both CC-3 and p27 protein expression was significantly upregulated in combination group compared to the control group (Fig. 7F). Taken together, these data indicated that targeting TWIST1 could serve as a potential therapeutic strategy to overcome MET driven resistance in oncogene-driven NSCLC.

## Discussion

Drug resistance is the major cause of the failure of clinical effectiveness of targeted therapy in NSCLC. Even though high response rates of MET TKIs in *MET* amplified and *MET* exon 14 skipping mutant NSCLC are observed [12–15, 47], acquired resistance is inevitable and the majority of patients with targetable *MET* aberrations fail to respond to these MET TKIs [17, 48]. Furthermore, recurrent non-exon 14 skipping *MET* mutations in the sema and catalytic domains are currently not targetable by available MET TKIs [49]. Clearly, new approaches to target MET-driven NSCLC are needed. In the current study, we have demonstrated that targeting the EMT-TF, TWIST1 may be a therapeutic strategy to enhance sensitivity to MET TKIs, overcome MET TKI resistance and potentially restore sensitivity to TKIs targeting other oncogenic drivers such as EGFR when *MET* alterations are the mechanism of acquired resistance. This has important therapeutic implications for other oncogene driven NSCLCs as *MET* amplification and HGF expression are common mechanisms of de novo and acquired resistance to TKIs in NSCLC [7, 40], including to the third generation EGFR TKI osimertinib (15–22%) [50, 51]. In addition, at the time of resistance to ALK TKIs in *ALK* fusion positive NSCLC, *MET* amplification along with activation of other signaling pathways is often observed [9, 11, 52]. Recent data has also identified *MET* amplification as a recurrent mechanism of resistance to targeted therapy in *RET* fusion subset of NSCLC as 15% of patients who develop resistance to RET inhibitors harbor acquired *MET* amplification without concurrent *RET* resistance mutations [53]. *MET* amplification has also been identified as a mediator of resistance in *NTRK* fusion positive tumor [54] and *KRAS G12C* mutant subtype of NSCLC [55, 56].

In addition to *MET* amplification as a mechanism of acquired resistance, high HGF levels have been observed in patients with de novo resistance (29%) and acquired resistance (~60%) [40, 41, 57]. Unfortunately, multiple attempts to target HGF or MET with monoclonal antibodies (Mab) in combination with TKIs in the clinic have failed due to toxicity and lack of efficacy [3, 58, 59]. Most notably, the combination of a MET Mab, onartuzumab (MetMab) with erlotinib was unsuccessful in both *EGFR* wild type and mutant patients who were selected on the basis of MET expression level [60, 61]. Conversely, previous reports have shown activity of MET TKIs in combination with EGFR TKIs in *MET* amplified *EGFR* mutant, EGFR TKI resistance tumors [29, 62, 63]. Since resistance to these combinations is inevitable, we tested a novel therapeutic strategy of dual TWIST1 and EGFR inhibition. Our data demonstrated that this combination significantly reduced tumor growth in this EGFR TKI resistant *EGFR* mutant *MET* amplified model (Fig. 8E). This suggests that further development of TWIST1 inhibitors is warranted as these would be expected to have broad activity against oncogenic driven NSCLC and specifically, both treatment naïve and MET TKI resistant MET driven tumors.

Since PDXs are directly established from patients and thus closely reproduce the genetic, molecular and histological heterogeneity of original tumor, in the present study, we generated capmatinib resistant PDXs and show that TWIST1 was significantly expressed at the time of *MET* TKI as well as in a separate *EGFR* TKI resistance PDX model (Fig. 1). We also demonstrated that HGF increases TWIST1 expression through a post-translational mechanism, as HGF treatment significantly increases TWIST1 protein half-life but did not affect the TWIST1 mRNA levels (Fig. 3H, Supplementary Fig. 4B) as previously reported in a canine mammary cell line [64]. This finding also helps to potentially resolve a question raised by our previous observation that TWIST1 could induce resistance in *EGFR* mutant NSCLC and TWIST1 was increased at the time of resistance in vivo after EGFR TKI treatment [27]. We previously didn’t have an explanation for the high TWIST1 levels that were observed at the time of resistance but in retrospect these may have been mediated by increased HGF-MET signaling likely through the ERK pathway. Previous studies have demonstrated that PKB/AKT and ERK pathways are activated by HGF-MET signaling [4] and can regulate TWIST1 function and stability [45, 65]. Phosphorylation of TWIST1 on serine 68 (S68) by MAPKs such p38, JNK, and ERK1/2 leads to increased protein stability by inhibiting its ubiquitination and proteasomal degradation [45]. Interestingly, we have found that an ERK inhibitor can degrade exogenous TWIST1 (Fig. 3G), suggesting that ERK may be required for HGF/MET-dependent TWIST1 expression. Furthermore, we have found that a point mutation at the ERK1/2 phosphorylation site (S68D)(Supplementary Fig. 4A) rescues TWIST1 degradation from MET TKI (Fig. 3G). These results suggest that an ERK inhibitor may be more effective in MET TKI resistance *MET* altered tumors with high TWIST1 expression which will be explored in future studies.

Although EMT-TFs have been implicated in TKI resistance [27, 66, 67], therapeutic strategies to target these EMT-TFs have been lacking. In the current study, we demonstrated that targeting a single EMT-TF was sufficient to inhibit MET-driven lung tumorigenesis and overcome MET TKI resistance as sustained expression or inhibition of TWIST1 alone was sufficient to either induce MET TKI resistance or sensitivity, respectively (Figs. 4 and 7). Importantly, it was able to sensitize MET altered cell lines that have previously been shown to be partially resistant to MET TKIs such the H596 cells [21, 36, 37]. We have found that TWIST1 can mediate resistance to MET TKIs in vitro and in vivo through suppression of p27 expression (Fig. 5) and inhibition of TWIST1 can restore sensitivity to multiple MET TKIs (Fig. 7). We have found that that TWIST likely mediates MET TKI resistance through reduction of p27 levels (Fig. 4J, K and Supplementary Fig. 5I) and silencing of p27 was sufficient to induce resistance to both MET and TWIST1 inhibitors (Fig. 5 and Supplementary Fig. 6). Furthermore, we have demonstrated that p27 is downregulated at the time of acquired resistance and our MET TKI acquired resistance cells (H1993 CR500) fail to induce p27 in the presence of a capmatinib or crizotinib (Fig. 6). The p27 protein is a negative regulator of cell proliferation and acts primarily by inhibiting cyclin-dependent kinases (CDKs). Interestingly decreased p27 levels have been associated with increased sensitivity to CDK4/6 inhibitors [68, 69]. Therefore, our findings suggest *MET* altered NSCLC tumors with high TWIST1 and low p27 expression maybe more sensitive to the combination of a CDK4/6 inhibitor alone or in combination with MET TKIs.

In summary, our study identifies TWIST1 as a critical mediator of MET TKI resistance in *MET* altered NSCLC both in HGF-dependent and HGF-independent settings (Fig. 8G). Moreover, we identified an effective combinatorial approach of harmine with TKIs to overcome MET TKI and EGFR TKI resistance. In conclusion, our findings provide preclinical rationale for development of more potent TWIST1 inhibitors and potentially future clinical trials with TWIST1 inhibitors in MET driven NSCLC.

## Materials and methods

### Cell lines and reagents

All human NSCLC cell lines (H1437, H596, H1993, H1648, H2073, HCC-827, H23, H460) and embryonic kidney cell line HEK 293T were procured from American Type Culture Collection (ATCC) and grown in media recommended by ATCC. 11–18 cells were obtained from Dr. Christine Lovly (Vanderbilt University) and cultured in the recommended media [70, 71]. FVBW-17 and FVB-HGF cells were derived from carcinogen induced lung tumors in wild-type FVB/N or transgenic mouse model with lung specific Hgf expression respectively [39] and this current publication as previously described (Supplementary Table S1). Cell lines were authenticated by autosomal short tandem repeat (STR) DNA profiling done at University of Arizona-Genetics core (UAGC). Cell lines were tested for mycoplasma contamination every 6 months using MycoAlert Detection Kit (Lonza). Cycloheximide (C4859), Doxycycline hydrochloride (D3072) and Harmine (286044-1G) was obtained from Sigma-Aldrich (St Louis, MS). Capmatinib (HY-13404) was obtained from MedChem Express (NJ, USA). Crizotinib (PF-02341066), Tepotinib (EMD 1214063), and Erlotinib (OSI-744) were procured form Selleck Chemicals (Houston, TX). The ERK inhibitor (LY3214996) was supplied by Eli Lilly and Company. Recombinant Human HGF (294-HGN-005/CF 5 µg) was purchased from R&D Systems (Minneapolis, MN). 4-(Methylnitrosoamino)-1-(3-pyridyl)-1-butanone (NNK) (M325750) was purchased from Toronto Research Chemicals (Toronto, ON) and was dissolved in 0.9% saline.

### Cell viability assays

For all cell viability experiments, NSCLC cell lines were seeded in 96 well plates at appropriate cell density and incubated in 5% CO_2_ at 37 °C in a humidified atmosphere for 24 h. Cells were subsequently treated with concentration gradient of either TWIST1 inhibitor or tyrosine kinase inhibitors (TKIs) for 48 and/or 72 h. For all TWIST1 knockdown experiments, NSCLC cell lines were seeded in 96 wells at appropriate cell density and were infected with lentivirus for 120 h. For all TKI sensitivity experiments, NSCLC cell lines were seeded in 96 wells at appropriate cell density and were infected with lentivirus for 24 h. Following 24 h of infection, lentivirus was replaced with normal media or media with TKIs for 48 or 72 h. Cell viability was assessed using CellTiter96 Aqueous One Solution Cell Proliferation Assay Kit (Promega) or CellTiter-Glo (Promega) according to the manufacturer’s protocol. IC_50_ values were calculated using Graphpad Prism Version 8.4.3 software. Data was analyzed as previously described [26].

### Lentivirus production, transduction, and preparation of stable cell lines

The lentiviral particles were created using a four-plasmid system and infected as according to the TRC Library Production and Performance Protocols, RNAi Consortium, Broad Institute [72], and as described previously [25–27]. Briefly, 2.5 × 10^6^ HEK 293T cells were seeded in T-25 flasks, following a 24 h post incubation period, cells were transduced with respective plasmid DNA with the help of polyethylenimine (PEI) to generate lentiviruses. The complete list of shRNAs for TWIST1 and p27 is provided in supplementary table S2. All other vector construct, primer sequences were the same as reported earlier. All constructs were sequence verified.

TWIST1 overexpressing stable cell lines and their respective control cell lines were made by infecting lentiviruses with polybrene and selecting the cells with 1 µg/mL puromycin. The knockdown stable p27 and their vector control (scrambled) stable cell lines were made by infecting the cells with lentivirus and selecting the cells with 500 µg/mL hygromycin. FVBW-17 and FVB-HGF GFP-luciferase expressing stable cell lines were made by selecting the cells with 2 µg/mL of puromycin.

### Immunoblot analysis

For western blot analysis, protein was isolated after appropriate treatment by using RIPA buffer. Protein was quantified, prepared, for western blots were performed as previously described [25–27]. Supplementary Table S3 contains details of primary antibodies used in this study. Target proteins were detected by chemiluminescence (ECL and ECL-plus reagent by GE Healthcare).

### Real time qPCR

The mRNA expression level of TWIST1 and p27 was measured using the primers as previously reported [26, 27, 73]. Briefly, total RNA was isolated using QIAprep RNeasy Kit (Qiagen) as per manufacturer’s instruction followed by reverse transcription into cDNA (Applied Biosystems kit). Real-time qPCR (Applied Biosystems StepOne RT-PCR system-PerkinElmer) was then performed to amplify cDNA using PowerUp SYBR Green Master Mix (PerkinElmer Applied Biosystems) or TaqMan Universal PCR Master Mix (PerkinElmer Applied Biosystems) according to the manufacturer’s protocol.

### Clonogenic assay

For the clonogenic assay, cells were seeded in 12 well plate. Each well had 10,000 cells. Next day cells were treated with varying doses of capmatinib for treatment experiments. After 3 weeks of post-incubation, colonies in each well were fixed in 10% formalin, stained with crystal violet, and visualized.

### Mouse xenograft experiments

All in vivo mouse xenograft experiments were carried out as per the NIH animal use guidelines and protocol approved by the Institutional Animal Care and Use Committee (IACUC) at the University of Pittsburgh. Flank tumors were established in 7-9-week-old female NOD/SCID mice (Jackson Laboratories) by subcutaneous injection of 1 × 10^6^ H1993 TWIST1 over expressing stable cell line or the control cell line. The cells were suspended in a total volume of 200 μL of 1:1 PBS and Matrigel (BD Biosciences, San Diego, CA). When the tumor volume reached 150-250 mm^3^, animals were randomly divided into control and treated groups (*n* = 6), Methyl cellulose (0.25% w/v), was used as vehicle control. Capmatinib was dissolved in 0.25% w/v methyl cellulose and administered by oral gavage five times a week for 4 weeks at an equivalent drugs dose of 5 mg/kg animal body weight. Tumor size was measured at regular intervals using a digital vernier caliper. At the end of study, tumors were excised, weighed, and images were taken.

For H596 Scr and H596 shTWIST1 experiments, H596 cell were infected and 1 × 10^6^ cells were implanted as similar to previously described for other cell lines [25]. Growth patterns were summarized graphically by plotting the mean and SD for each treatment group over time.

### Transgenic mice

All mice were maintained in pathogen-free animal facilities and experiments were conducted under an approved Institutional Animal Care and Use Committee protocol at the University of Pittsburgh (Pittsburgh, PA). The mice were caged in pathogen-free facilities, with no more than five per cage with controlled atmosphere and free access to water and food. NNK (4-(methylnitrosoamino)-1-(3-pyridyl)-1-butanone) induced constitutive Hgf and inducible Twist1 transgenic mice in the FVB/N inbred background were of the genotype: **CTH** (***C****CSP-rtTA*/***T****wist1-tetO-luc*/*CCSP-****H****gf*) *CCSP-rtTA/* *Twist1-tetO-luc /CCSP-Hgf* (CTH) created by crossing *CCSP-Hgf* (CH) mice with *CCSP-rtTA/Twist1-tetO-luc* (CT). CTH mice constitutively overexpress HGF and doxycycline inducibly express Twist1. All the mice were weaned 3–4 weeks of age and then placed on doxycycline containing chow or control chow at 6 weeks of age. After two weeks, both group of mice received NNK twice per week (3 mg per i.p. injection) for 5 weeks (6 mg/week). Following NNK treatment, mice were observed for 17 weeks and then harvested for tumor analysis. Lung tumors were analyzed by formalin inflation of the lungs and gross examination using a dissection microscope and Motic Image software to count and measure surface tumors [38]. In a separate cohort, all mice received doxycycline and NNK at the indicated doses above and then at 17 weeks after NNK, half of the mice were randomized to continue doxycycline (Twist1 ON) and half were placed on regular chow (Twist1 OFF) for four weeks at which point the lungs were harvested for tumor analysis as above. Lungs were harvested at two weeks after doxycycline withdrawal in a subset of animals that were used for IHC studies.

### Patient derived xenograft (PDX)

For the generation of capmatinib resistant PDXs, 6–8 week old athymic nude mice were used to implant an established MET TKI sensitive PDX (UW-21 lung) from a patient harboring a *MET* exon 14 mutant NSCLC tumor [28]. The tumor samples were cut into 3–4 mm pieces and implanted into mice subcutaneously. The xenografts were allowed to grow for 6–8 weeks. Mice were then treated for 3–4 weeks with capmatinib at an equivalent dose of 5 mg/kg animal body weight by oral gavage, 5 times in a week. The tumor responded completely and but later regrew. At time of regrowth, the tumor was again treated with capmatinib for the three weeks. This cycle was repeated till tumor became non-responsive or resistant to capmatinib.

The *EGFR* mutant *MET* amplified PDX established from the lung mass (MSK-LX29) of a patient with *EGFR* mutant (*L858R* mutation) with an acquired *MET* amplification after progression on erlotinib [29]. Briefly, tumor samples were cut into approximately 2 × 2 × 2 mm^3^ pieces and transplanted into 6–8-week-old athymic nude mice [Crl:NU (NCr)-F] (Charles Rivers). The mice were maintained at the animal house facility according to the ethical approval obtained for the establishment of PDXs. Once PDX hind flank tumors reached ≥150 mm^3^, animals were randomized into four groups (n = 7), control (vehicle), harmine, erlotinib and erlotinib + harmine group. The drugs were administered by via intraperitoneal injection daily, 5 days a week for 16 days. The doses of drugs were as follows: Control group (PBS), harmine group (10 mg/kg), erlotinib group (25 mg/kg) and harmine + erlotinib group (10 mg/kg harmine + 25 mg/kg erlotinib). Tumor sizes [1/2(length × width 2)] were measured by digital caliper twice a week until they reached ~ 2000 mm^3^. When this size was reached, animals were sacrificed, and tumors were collected for further analysis. We generated the BM 12-15 PDX as previously described [26, 30]. All other PDXs (BM 04-14, BM 18-17, BM 15-16, BM 16-16, BM 20-17, BM 11-15, BM 08-15 and BM 07-15) were established from resected brain metastasis on an IRB approved protocol at the University of Pittsburgh Medical Center. 2 mm^2^ tumor tissues cut with sterile blade were implanted subcutaneously as previously described [30].

### Fluorescent in situ hybridization (FISH) and Immuno-histochemistry (IHC)

FISH was performed on formalin-fixed, paraffin-embedded tissue sections in accordance with the manufacturer’s instructions by a CLIA certified laboratory (UPMC Presbyterian Department of Anatomic Pathology, In situ Hybridization Laboratory). Unstained sections were placed on electrostatically charged slides and then evaluated *MET* (7q31) and centromere 7q (CEP7q) ratio by using Kreatech MET/SE 7 probe. The tissues were scored by evaluating a total of 60 cells.

IHC and histology were performed as described previously [27, 30, 38, 74, 75]. Primary antibodies were used at the following dilutions: Ki-67 at 1:2000, cleaved caspase-3 at 1:500 and p27 at 1:250. Positively stained cells were counted in three random fields (20X) and mean was taken for quantitation.

### Statistical analysis

Student t-test, ANOVA with Tukey’s multiple comparison testing, was performed where indicated. For xenograft experiments, growth patterns were summarized graphically by plotting the mean and SD for each treatment group over time. A conditional t test is used to compare shTWIST1 tumors with the control (shScr) or TWIST1 over expressing tumor with control tumor (W118Δ). *P* < 0.05 is considered statistically significant. For transgenic animal studies, we used cohorts of >15 animals each. This was based on sample size calculations assuming experimental condition will result in animals that have a mean survival that is 45% longer than control treated mice with a power of 80% to detect a difference using the Kaplan–Meier long-rank test. For PDX experiment, a student t test was performed on the finalized tumor volumes between the control and treated groups. A conditional t test is used to compare shTWIST1 tumors with the control (shScram). *P* < 0.05 is considered statistically significant.

### Study approval

Patient derived xenografts were obtained from patients undergoing standard of care craniotomies after informed consent to University of Pittsburgh IRB protocol #19080321. All animal studies were approved and performed under IACUC protocol #21089597.

### Supplementary information

Supplementary File 1

Supplementary File 2

Supplementary File 3

## Data Availability

All data are available in the main text or the supplementary materials.

## Materials availability

Cells described in this manuscript that are not publicly available from the ATCC, are available through a materials transfer agreement (MTA).
