# Supplementary material for: TWIST1 is a critical downstream target of the HGF/MET pathway and is required for MET driven acquired resistance in oncogene driven lung cancer
Source: Oncogene. 2024 Mar 1;43(19):1431–44. doi: 10.1038/s41388-024-02987-5 (PMC11068584; doi:10.1038/s41388-024-02987-5)
Supplement: Supplementary file 1 [file 41388_2024_2987_MOESM1_ESM.pdf]

Supplementary fig. legends.

Supplementary Fig. 1: TWIST1 is required in *MET* altered NSCLC *in vitro.* (A) fluorescent in situ hybridization (FISH) of BM 16-16 PDXs. Silencing of TWIST1 leads to growth inhibition in *MET* altered NSCLC cell lines. shRNA silencing of TWIST1 lead to growth inhibition as demonstrated in triplicates by crystal violet staining in **(B)** *MET* amplified H1648 cell lines and **(C)** *MET* mutant H596 cell lines. The cells were infected with the indicated shRNAs, plated on Day 4 and stained after 15 days following infection.

Supplementary Fig. 2: IC50s of MET TKIs in *MET* altered cell lines. Cell viability assays in the indicated MET altered cell lines (H596, H1437 and H1993) 72 hours after treatment with the indicated MET TKIs (Capmatinib, Crizotinib and Tepotinib).

Supplementary Fig. 3: TWIST1 is required for HGF-MET driven NSCLC genetically engineered mouse model. In NNK induced ***C***_CSP-rtTA/_ ***T***_wist1-tetO-luc /CCSP-_***H***_gf_ (**CTH**) lung cancer model, TWIST1 expression leads to increased number of tumor in both male and female mice **(A)** but not the tumor size **(B)**. Data represent mean ±SEM. *, p<0.05, **, p<0.01. 2-tailed Student's t-test. NS: non significant.

Supplementary Fig. 4: TWIST1 mRNA expression after HGF treatment. (A) Illustration of the key domains and ERK phosphorylation site in the TWIST1 protein. **(B)** *TWIST1* mRNA expression was measured by quantitative real time PCR. The HGF treatment does not changes the TWIST1 mRNA expression.

Supplementary Fig. 5: TWIST1 over expression induces MET TKI resistance *in vitro.* (A) Cell-Titer Glo assay demonstrating that the H1993 TRE3G-TWIST1 cells remain sensitive to capmatinib due to TWIST1 degradation by capmatinib. **(B)** Western blot demonstrating complete loss of TWIST1 expression and induction of p27 in the

presence of 48 hours of capmatinib treatment. **(C-H)** TWIST1 over expression induces MET TKI resistance. Cell-Titer Glo assay demonstrating that TWIST1 overexpression from a CMV promoter decreases response to the indicated MET TKIs in **(C&D)** H1437 **(E&F)** H596 and **(G&H)** H1993 cell lines. Data represent mean ± SD (n = 4 technical replicates). *P < 0.05, **P < 0.01, ***P < 0.001,**** P < 0.0001, two-way ANOVA. **(I)** Western blot analysis demonstrating decreased MET TKI induction of p27 in TWIST1 overexpressing H1437 cell lines after MET TKI treatment. Colony formation assay with triplicates shown demonstrating that TWIST1 overexpression decreases response to TKIs in **(J)** H1437 **(K)** H596 and **(L)** H1993 cell lines.

Supplementary Fig. 6: p27 is required for MET TKI induced cytotoxicity. (A) Western blot demonstrating knockdown of p27 with a second shRNAs in H1993 cells. Encircled in red box is the oligo which has better knockdown (shp27-2 vs shp27-3). **(B-E)** Cell-Titer Glo assay demonstrating that p27 partial knockdown with shp27-3 decreases response to **(B)** capmatinib **(C)** crizotinib **(D)** tepotinib. and **(E)** harmine. Data represent mean ± SD (n = 4 technical replicates). *P < 0.05, **P < 0.01, ***P < 0.001,**** P < 0.0001, two-way ANOVA. **(F)** Clonogenic assay demonstrating silencing of p27 in *MET* amplified H1993 cells leads cells to capmatinib resistance compared to control cells (Scr) as demonstrated in triplicates by crystal violet staining. The cells were treated with the indicated doses of capmatinib and stained after 15 days following treatment.

Supplementary Fig. 7: TWIST1 inhibition sensitizes to MET TKIs in *MET* altered NSCLC. (A-F) Cell viability assay demonstrating that genetic inhibition of Twist1 with shRNA overcomes resistance to crizotinib and capmatinib in H1437 **(A&B)**, H596 **(C&D)** and H1648 **(E&F)** cell lines respectively. **(G)** Western blot demonstrating induction of p27

after Twist1 inhibition with shRNAs. Encircled in red boxes are the panel of p27 expression which has maximum fold change (Scr vs sh TWIST1-2 and sh TWIST1). Data represent mean ± SD (n = 4 technical replicates). *P < 0.05, **P < 0.01, ***P < 0.001,**** P < 0.0001, two-way ANOVA.

Supplementary Table S1. Cell lines used in this study.

Cell line	Genomic alteration/status	Histopathology	Tumor source
H2073	*MET* wild type	adenocarcinoma	primary
H1993	*MET* amplified	adenocarcinoma	metastasis
H1648	*MET* amplified	adenocarcinoma	metastasis
H596	*MET* exon 14 skipping mutation	Adeno-squamous carcinoma	primary
H1437	*MET* mutant	adenocarcinoma	metastasis
H23	Kras G12C mutation	adenocarcinoma	primary
11-18	EGFR L858R mutation	adenocarcinoma	primary
HCC-827	EGFR-ΔE746-A750	adenocarcinoma	primary
H460	Kras mutation	large cell carcinoma	metastasis
FVBW-17	Kras mutation	adenocarcinoma	mouse
FVBCH-17	HGF overexpressing	adenocarcinoma	mouse

Supplementary Table S2. Sequences for TWIST1 and p27 shRNA (5'-3') used in this study.

shRNA Target	Clone ID	Target sequence	shRNA sequence
shTWIST1-1	TRCN0000020539	GCATTCTGATAG AAGTCTGAA	CCGGGCATTCTGATAGAAGTC TGAACTCGAGTTCAGACTTCT ATCAGAATGCTTTTT
shTWIST1-2	TRCN0000020540	CCTGAGCAACAG CGAGGAAGA	CCGGCCTGAGCAACAGCGAG GAAGACTCGAGTCTTCCTCGC TGTTGCTCAGGTTTTT
shTWIST1-3	TRCN0000020543	GCTGGACTCCAA GATGGCAAG	CCGGGCTGGACTCCAAGATG GCAAGCTCGAGCTTGCCATCTTGGAGTCCAGCTTTTT
shp27-1	TRCN0000009857	GAATGGTGATCA CTCCAGGTA	CCGGAATGGTGATCACTCCAGGTACTCGAGTACCTGG AGTGATCACCATTCTTTTTG
shp27-2	TRCN0000039932	CAGCGCAAGTGG AATTTCGAT	CCGGCAGCGCAAGTGGAATTTCGATCTCGAGATCGAA ATTCCACTTGCGCTGTTTTTG
shp27-3	TRCN0000009858	CACACTTGTAGG ATAAGTGAA	CCGGCACACTTGTAGGATAAGTGAACTCGAGTTCACT TATCCTACAAGTGTGTTTTTG

Supplementary Table S3: List of primary antibodies used in current study.

Antibody	Company	Catalogue number	Dilution
Cleaved PARP	Cell Signaling Technology	5625	1:250
E-Cadherin	Cell Signaling Technology	3195	1:1000
Vimentin	Cell Signaling Technology	5741	1:1000
TWIST1	Abcam	ab50887	1:200
GAPDH	Santa Cruz	sc-25778	1:1000
Actin	Millipore	MAB1501	1:10,000
p27	Abcam	ab32034	1:1000
p21	Millipore	630870	1:1000
E2A	Santa Cruz	sc-762	1:200
