# Supplementary material for: TWIST1 is a critical downstream target of the HGF/MET pathway and is required for MET driven acquired resistance in oncogene driven lung cancer
Source: Oncogene. 2024 Mar 1;43(19):1431–44. doi: 10.1038/s41388-024-02987-5 (PMC11068584; doi:10.1038/s41388-024-02987-5)
Supplement: Supplementary file 2 [file 41388_2024_2987_MOESM2_ESM.pptx]

## Slide 1
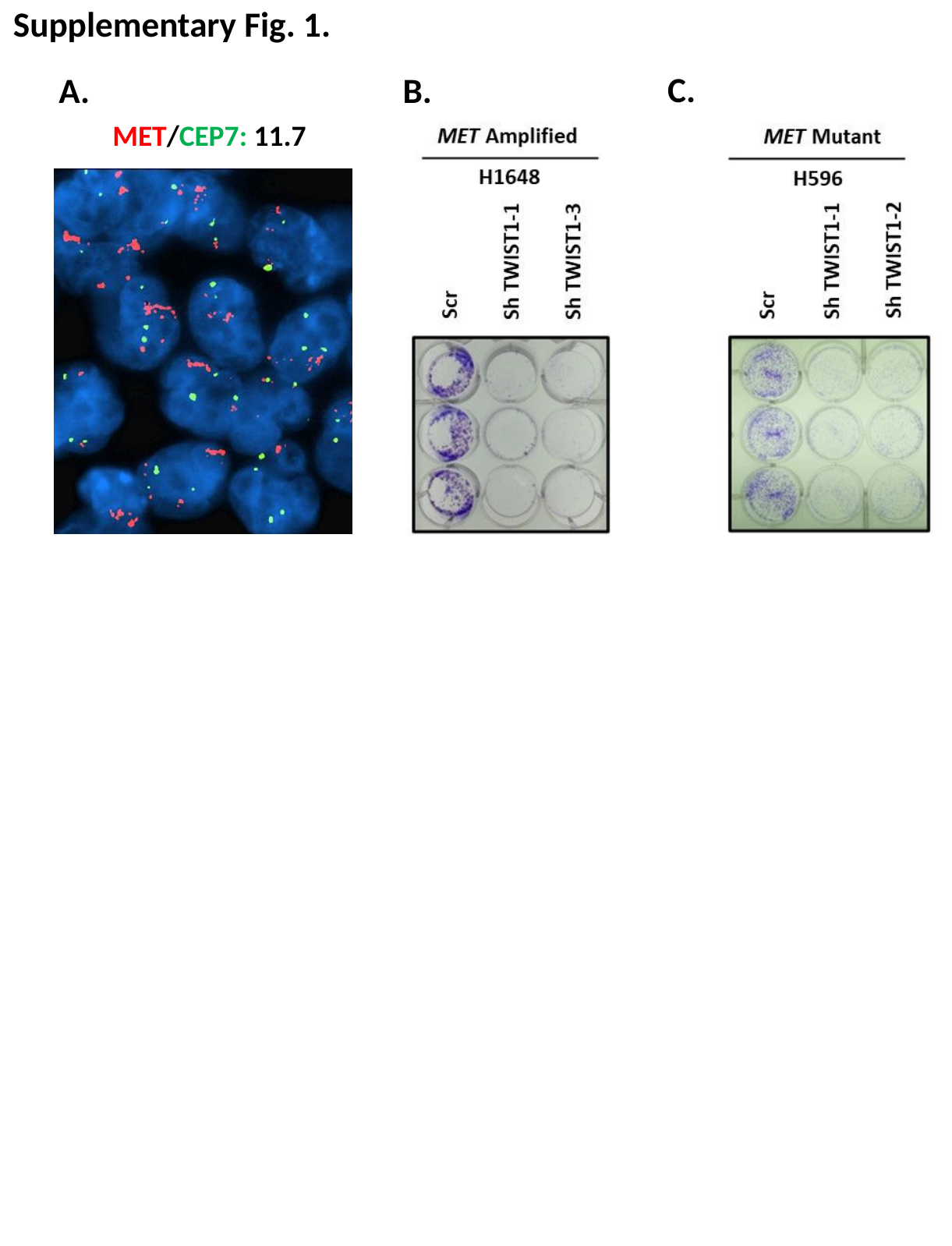

Supplementary Fig. 1.
C.
A.
B.
MET/CEP7: 11.7

## Slide 2
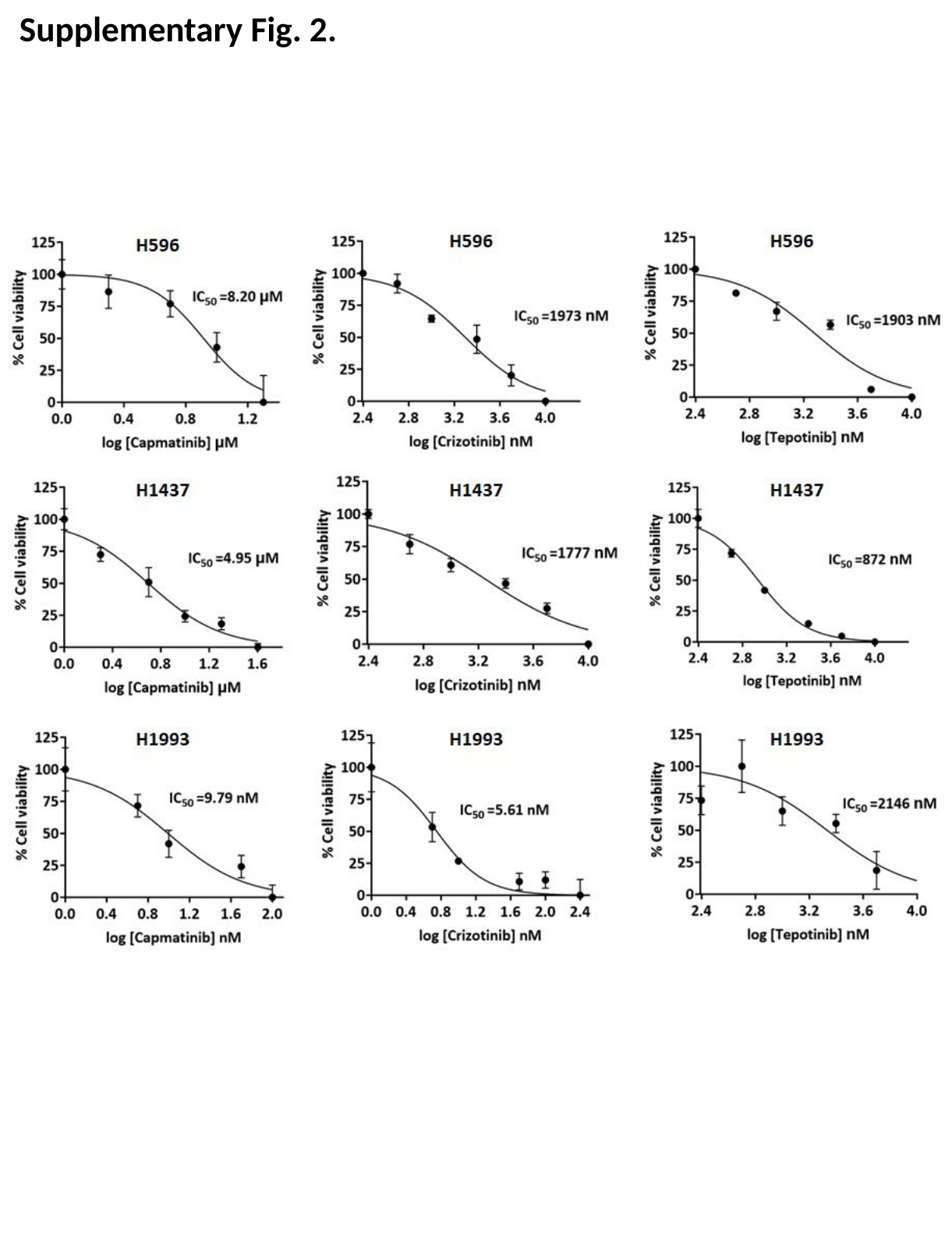

Supplementary Fig. 2.

## Slide 3
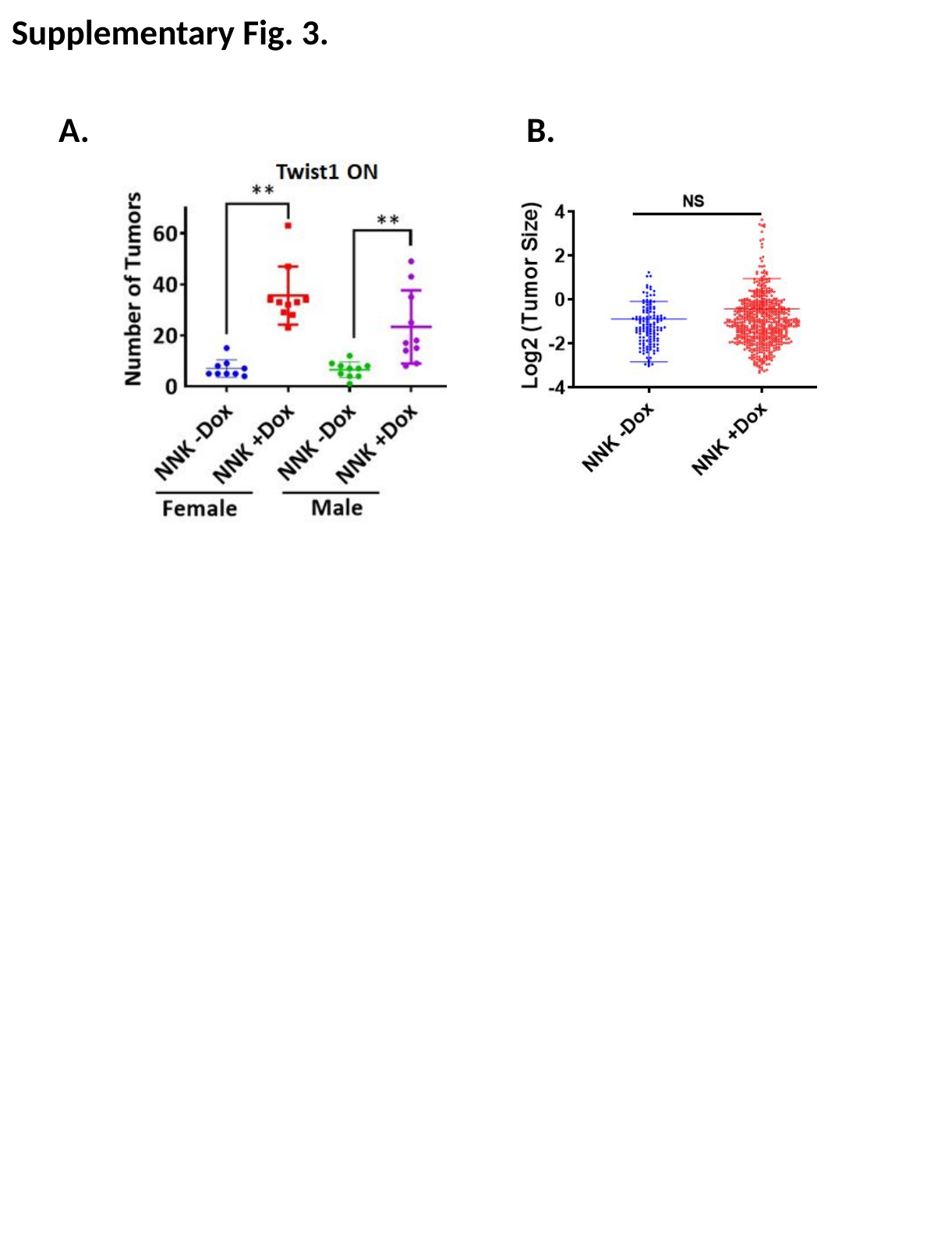

Supplementary Fig. 3.
A.
B.

## Slide 4
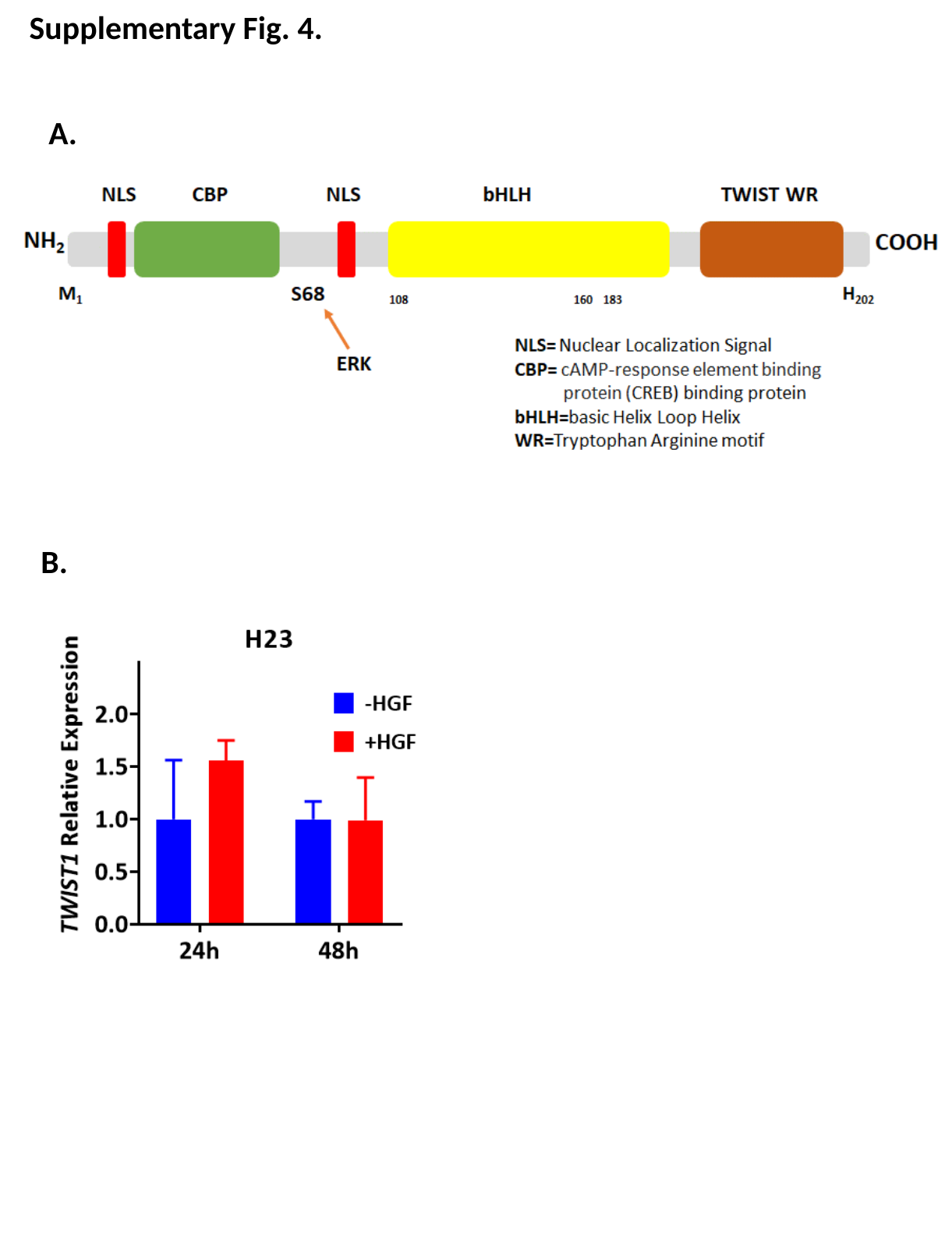

Supplementary Fig. 4.
A.
B.

## Slide 5
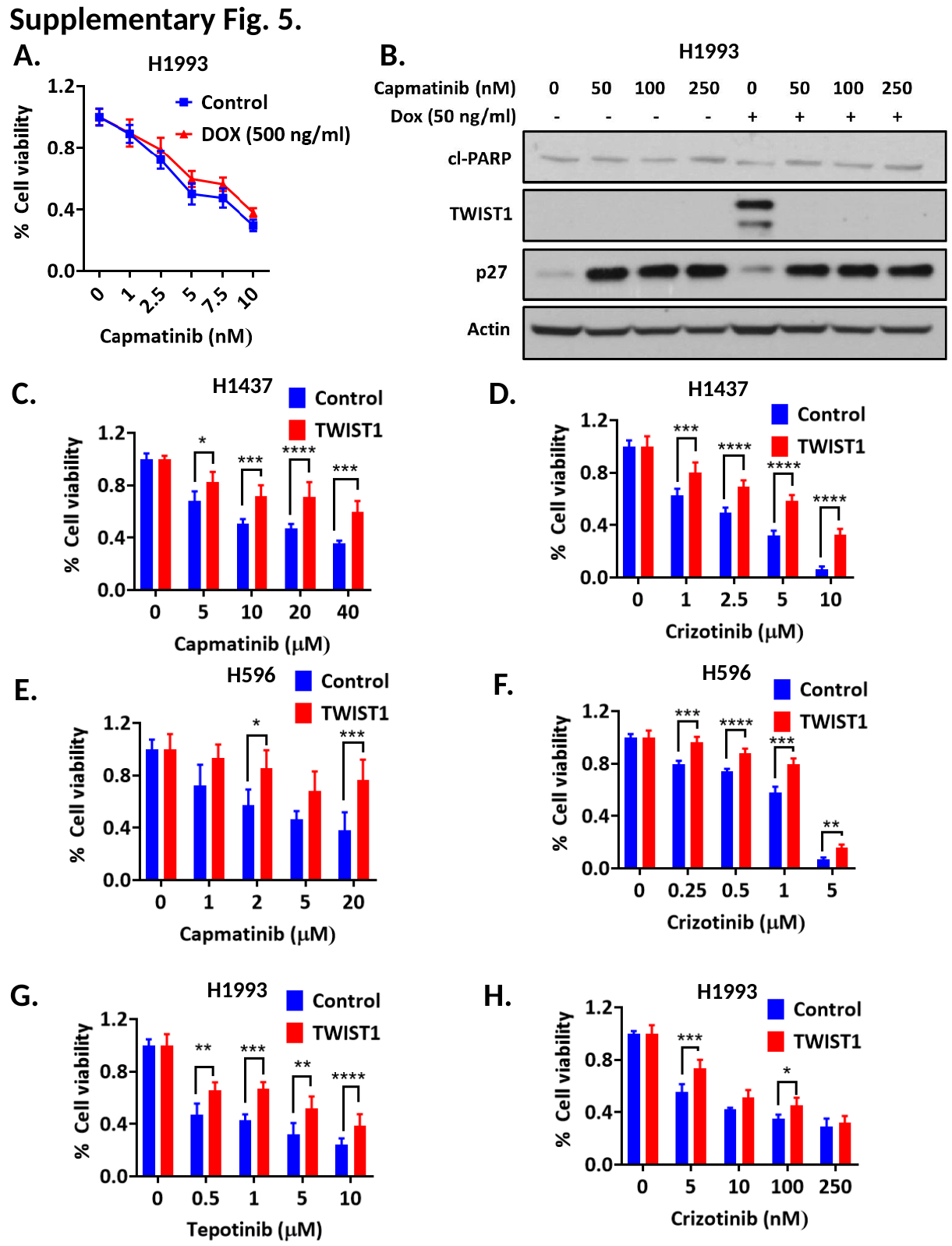

Supplementary Fig. 5.
B.
A.
H1993
H1993
H1437
C.
D.
H1437
H596
H596
F.
E.
G.
H.
H1993
H1993

## Slide 6
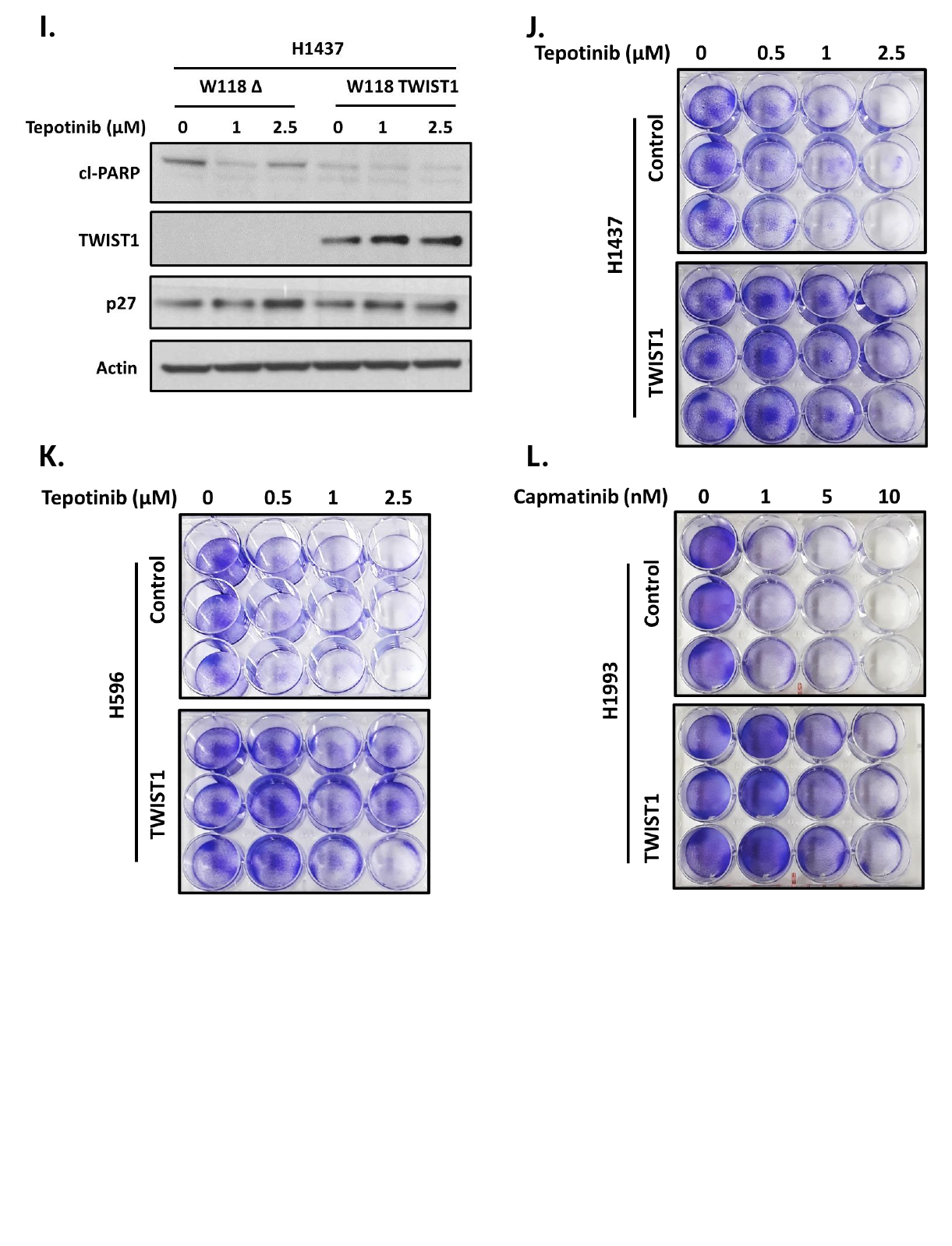

I.
J.
L.
K.

## Slide 7
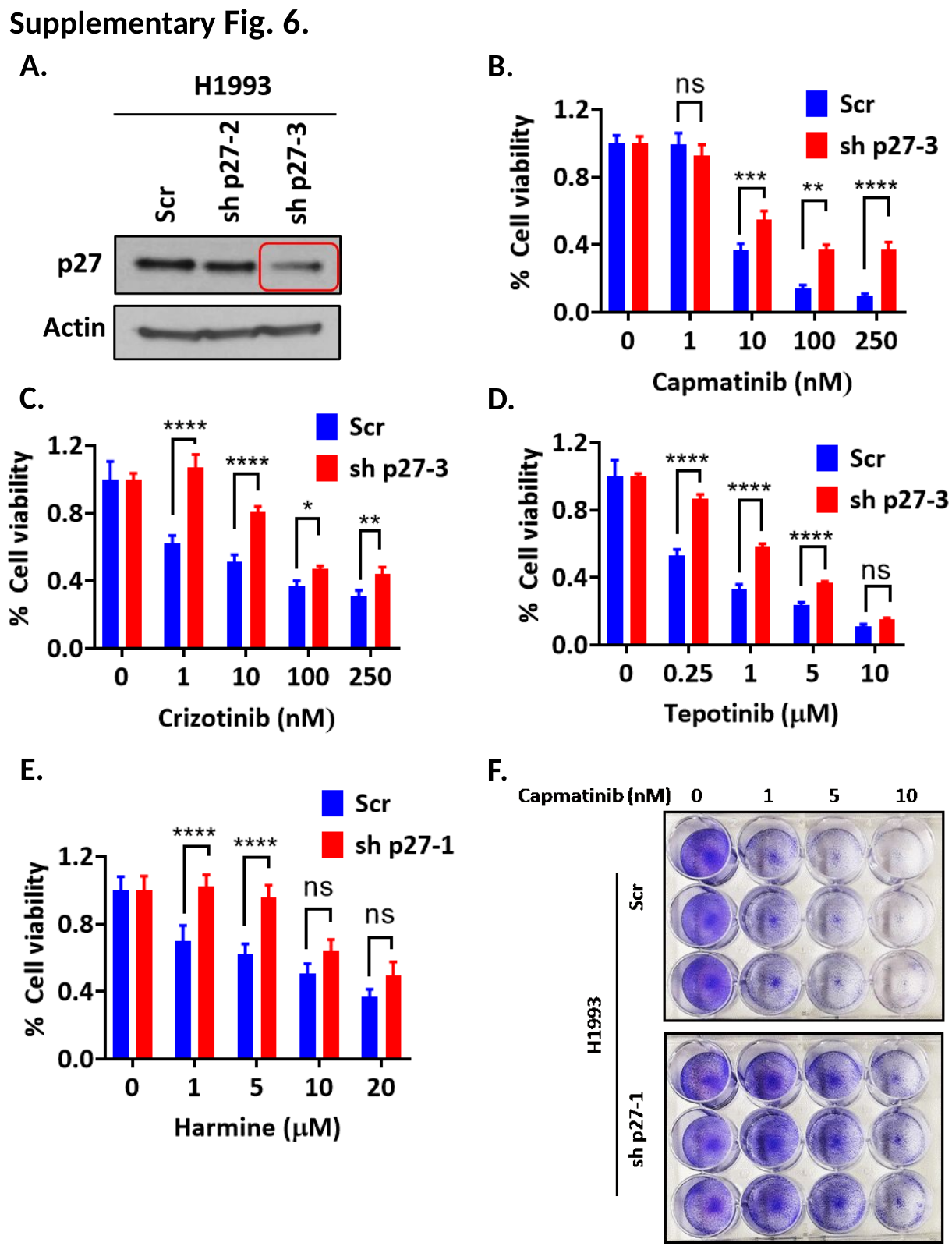

Supplementary Fig. 6.
A.
B.
C.
D.
E.
F.

## Slide 8
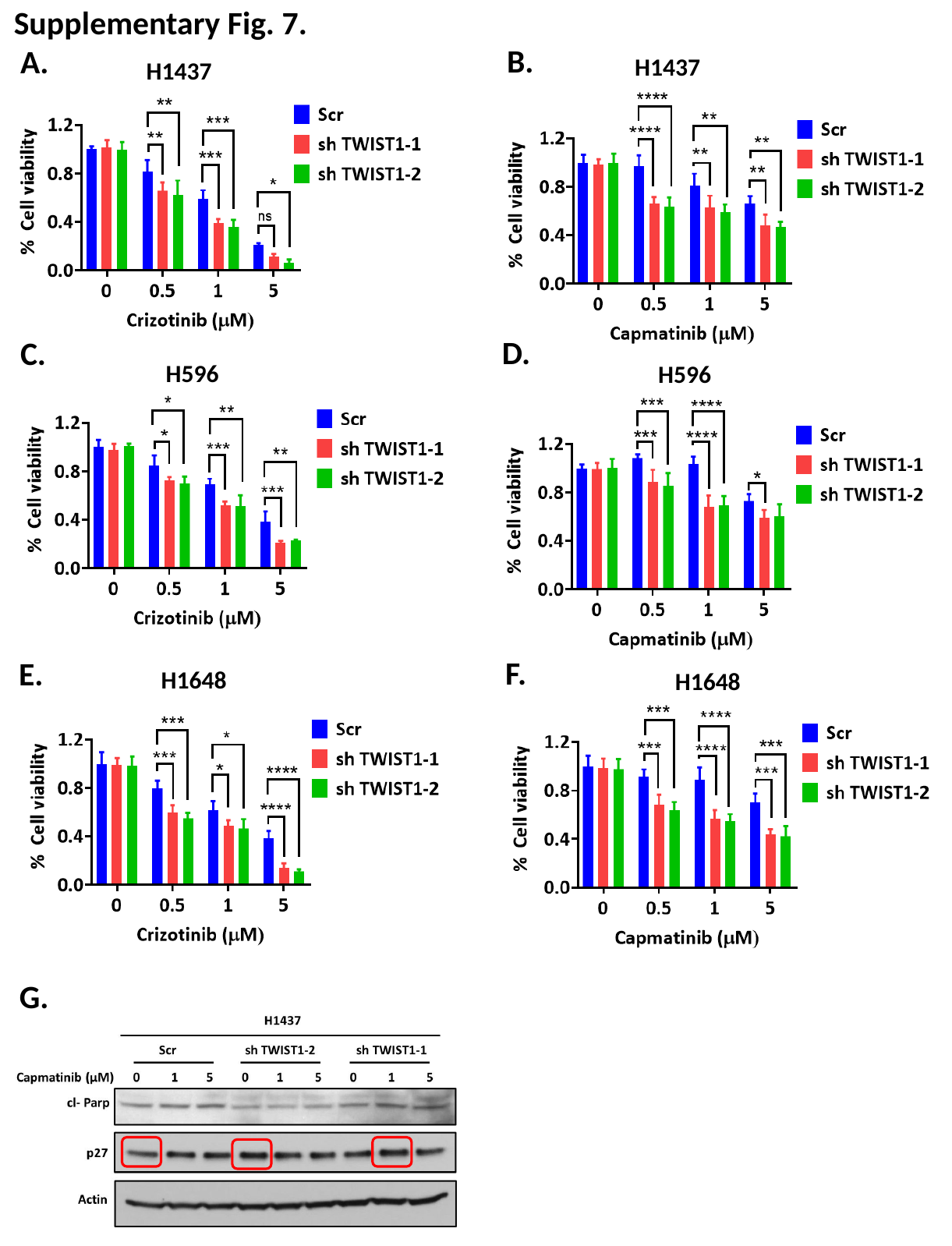

Supplementary Fig. 7.
B.
A.
H1437
H1437
D.
C.
H596
H596
F.
E.
H1648
H1648
G.
