# Supplementary material for: TWIST1 is a critical downstream target of the HGF/MET pathway and is required for MET driven acquired resistance in oncogene driven lung cancer
Source: Oncogene. 2024 Mar 1;43(19):1431–44. doi: 10.1038/s41388-024-02987-5 (PMC11068584; doi:10.1038/s41388-024-02987-5)
Supplement: Supplementary file 3 [file 41388_2024_2987_MOESM3_ESM.pptx]

## Slide 1
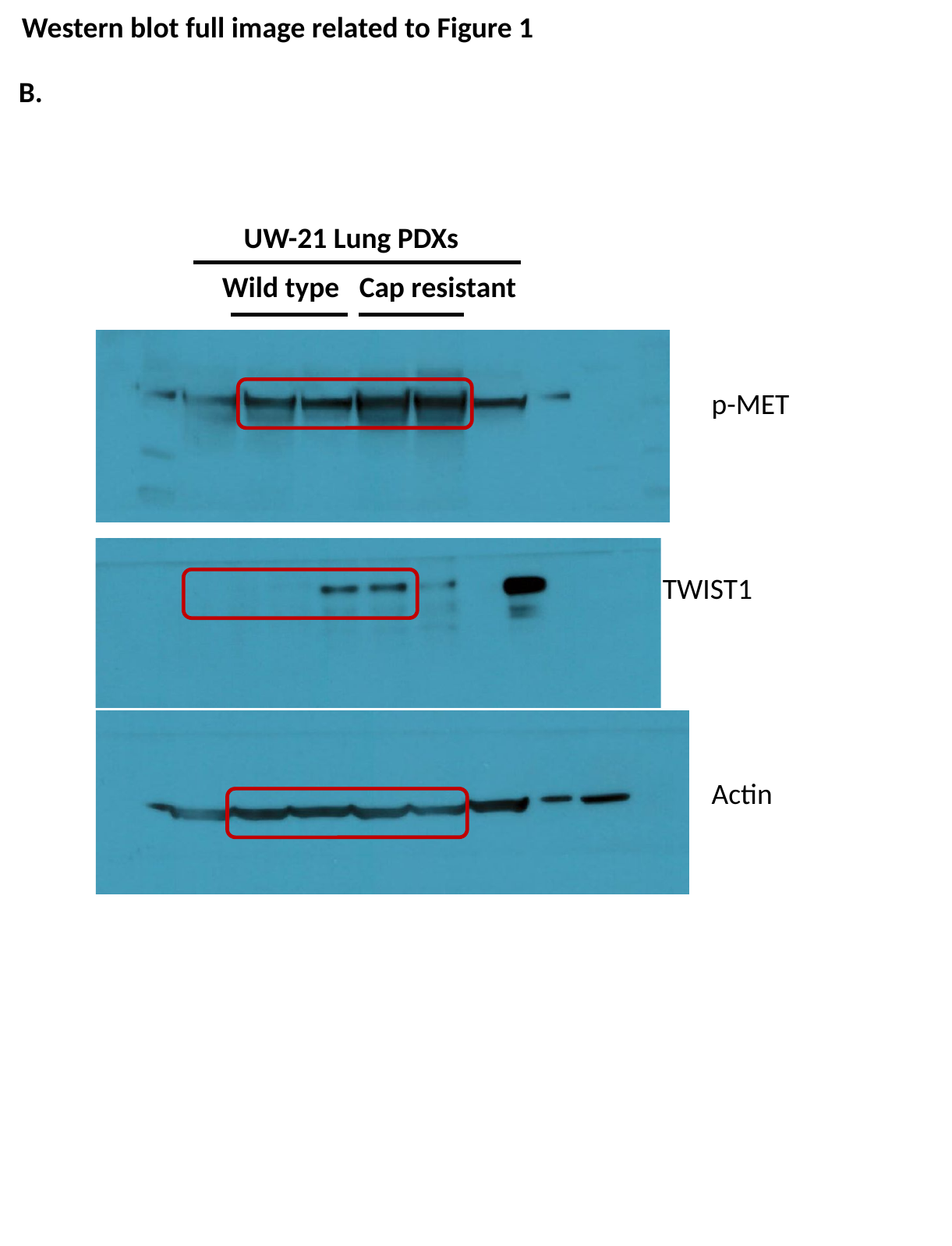

Western blot full image related to Figure 1
B.
UW-21 Lung PDXs
 Wild type Cap resistant
p-MET
TWIST1
Actin

## Slide 2
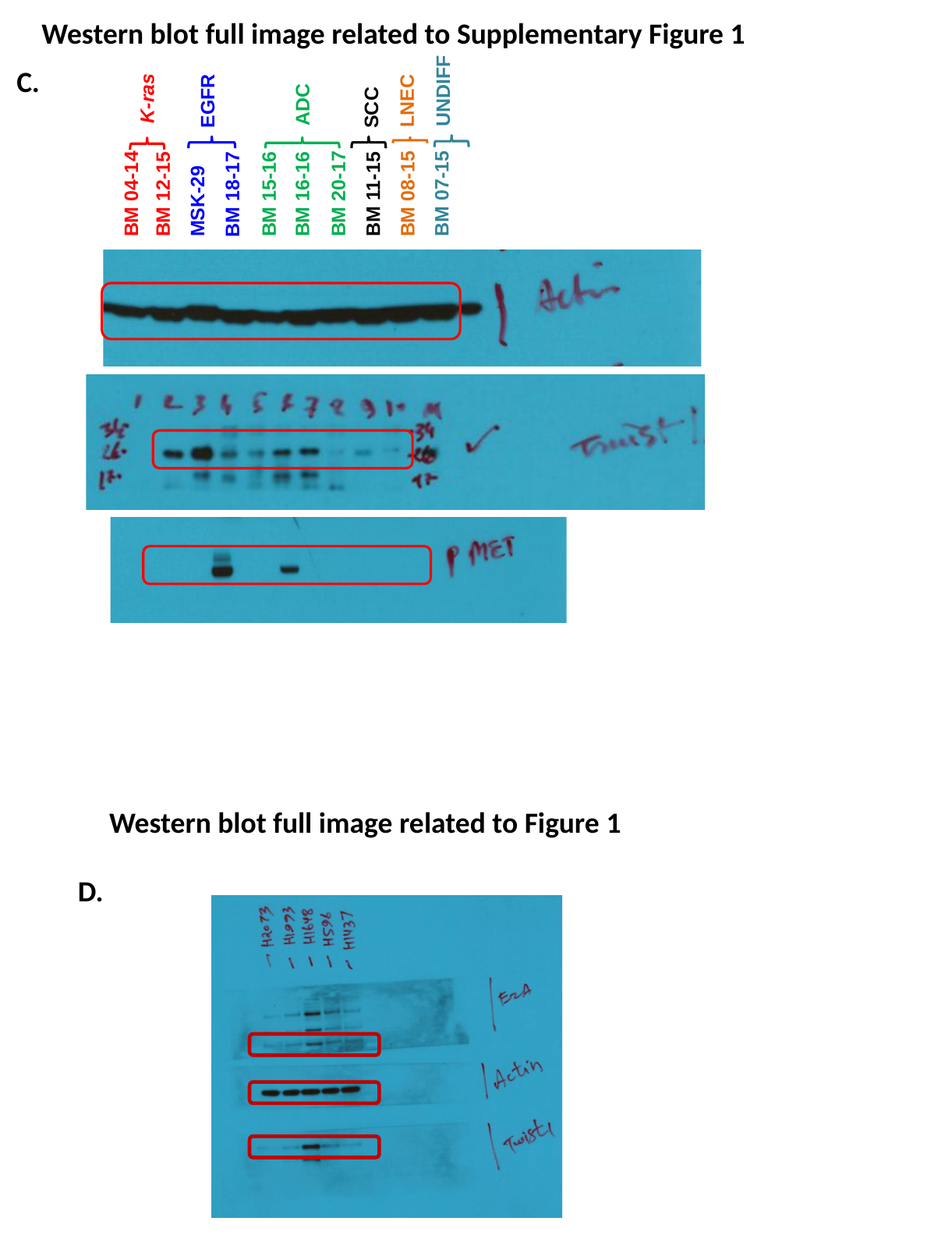

Western blot full image related to Supplementary Figure 1
UNDIFF
K-ras
LNEC
EGFR
ADC
SCC
BM 07-15
BM 11-15
BM 20-17
BM 16-16
BM 12-15
BM 15-16
BM 04-14
BM 08-15
BM 18-17
MSK-29
C.
Western blot full image related to Figure 1
D.

## Slide 3
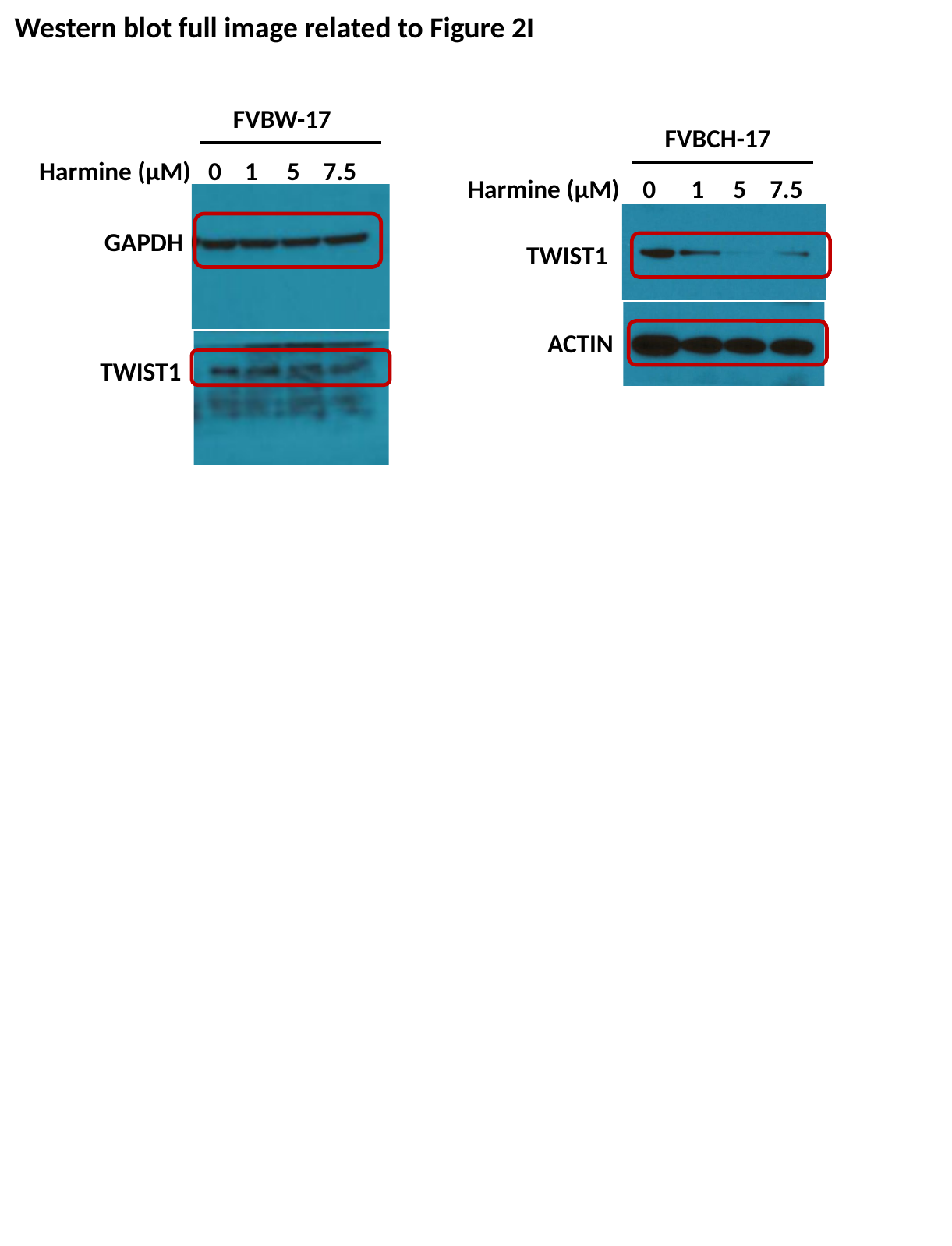

Western blot full image related to Figure 2I
FVBW-17
Harmine (µM) 0 1 5 7.5
GAPDH
TWIST1
FVBCH-17
Harmine (µM) 0 1 5 7.5
TWIST1
ACTIN

## Slide 4
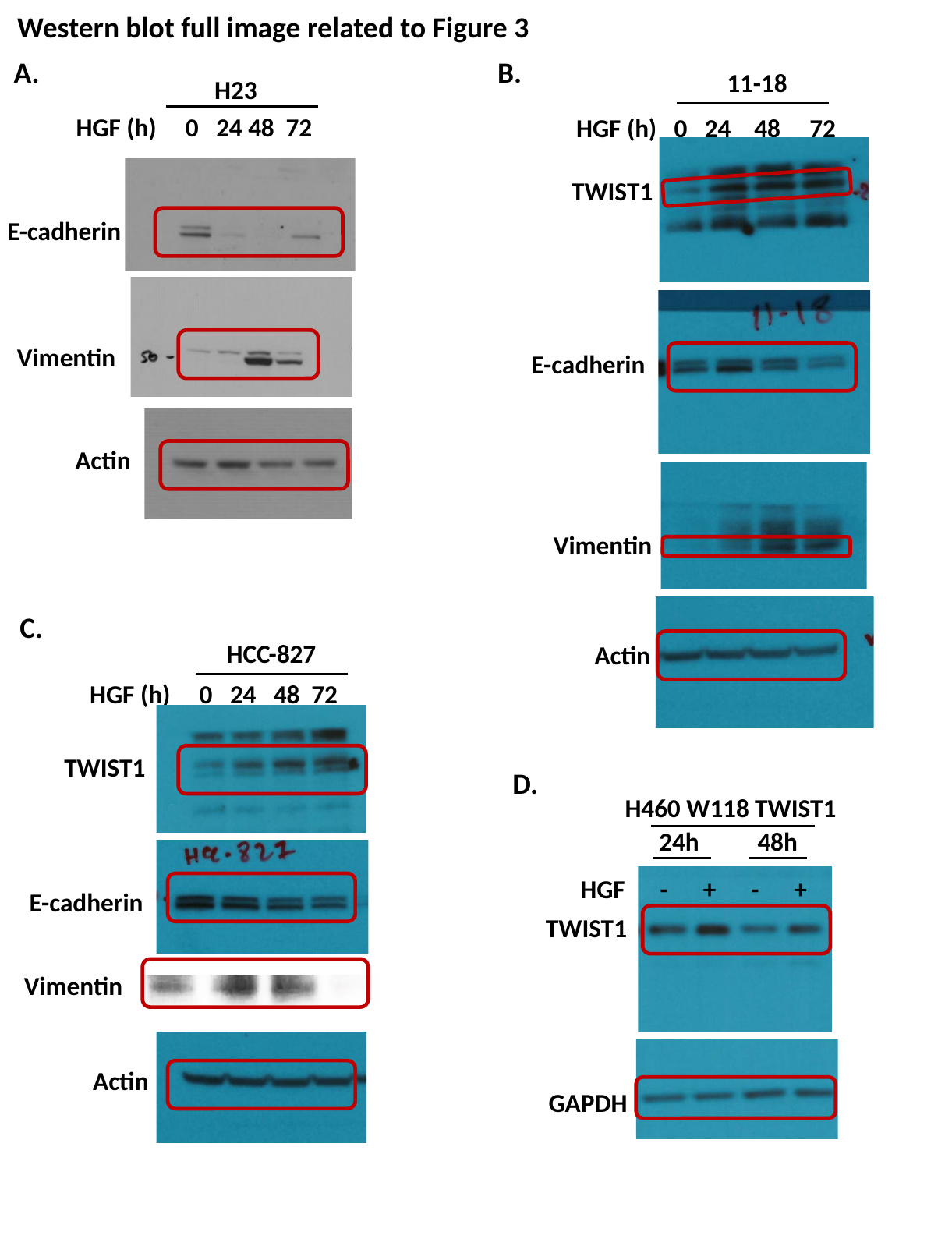

Western blot full image related to Figure 3
A.
B.
11-18
HGF (h) 0 24 48 72
TWIST1
E-cadherin
Vimentin
Actin
H23
HGF (h) 0 24 48 72
E-cadherin
Vimentin
Actin
C.
HCC-827
HGF (h) 0 24 48 72
TWIST1
D.
H460 W118 TWIST1
24h 48h
HGF - + - +
TWIST1
GAPDH
E-cadherin
Vimentin
Actin

## Slide 5
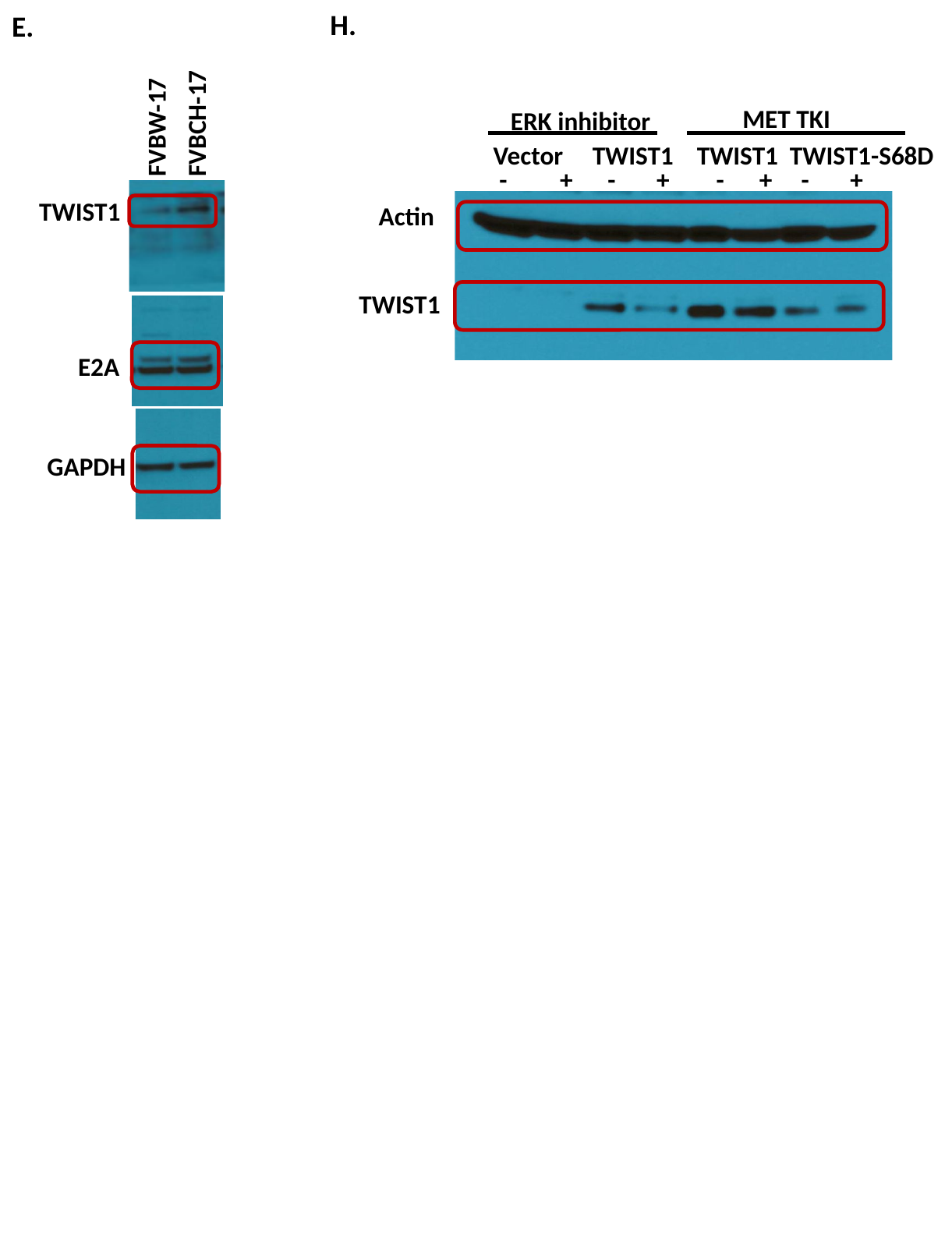

H.
MET TKI
ERK inhibitor
Vector TWIST1 TWIST1 TWIST1-S68D
- + - + - + - +
Actin
TWIST1
E.
FVBW-17
FVBCH-17
TWIST1
E2A
GAPDH

## Slide 6
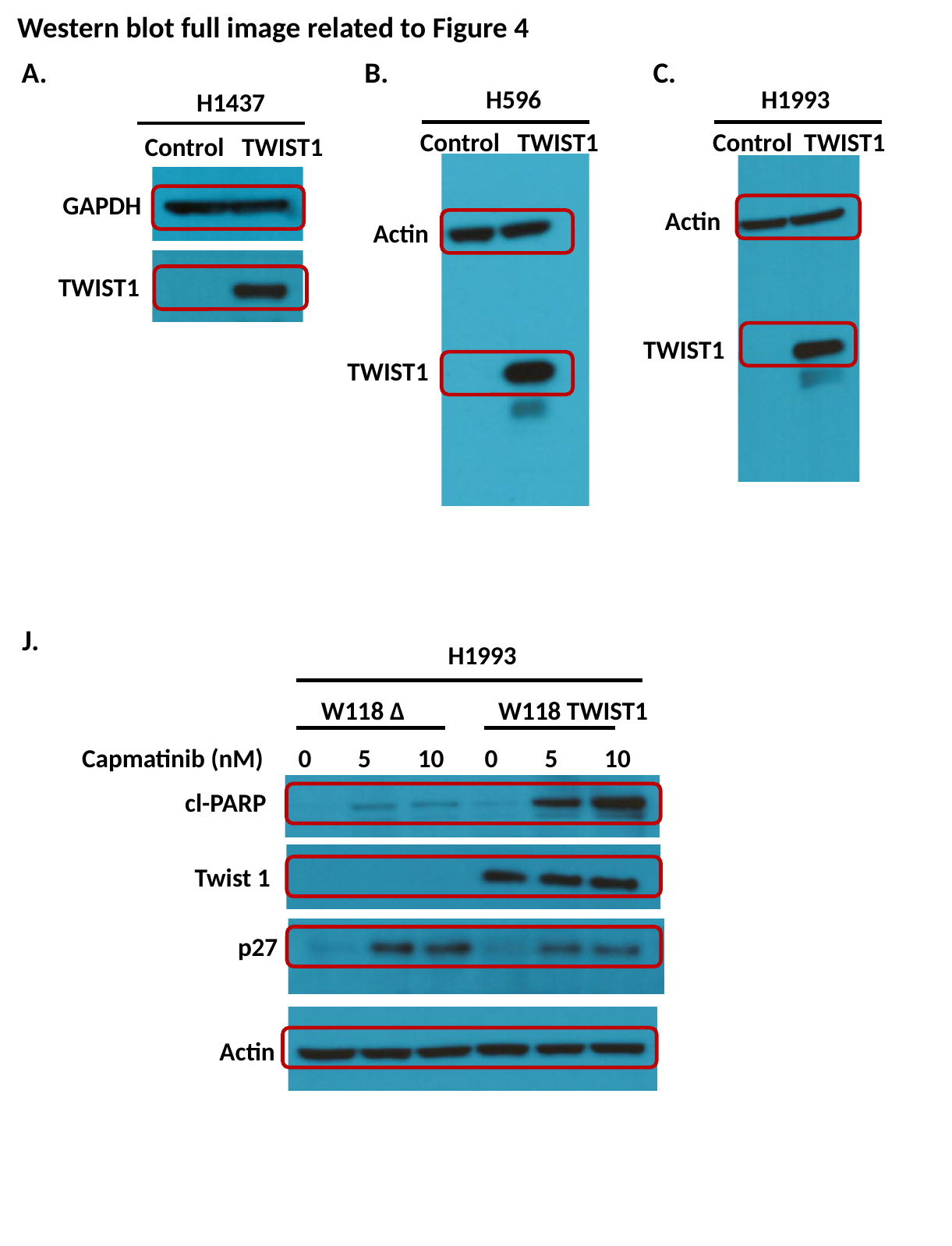

Western blot full image related to Figure 4
A. B. C.
H596
Control TWIST1
Actin
TWIST1
H1993
Control TWIST1
Actin
TWIST1
H1437
Control TWIST1
GAPDH
TWIST1
J.
H1993
 W118 Δ W118 TWIST1
cl-PARP
Twist 1
p27
Actin
 Capmatinib (nM) 0 5 10 0 5 10

## Slide 7
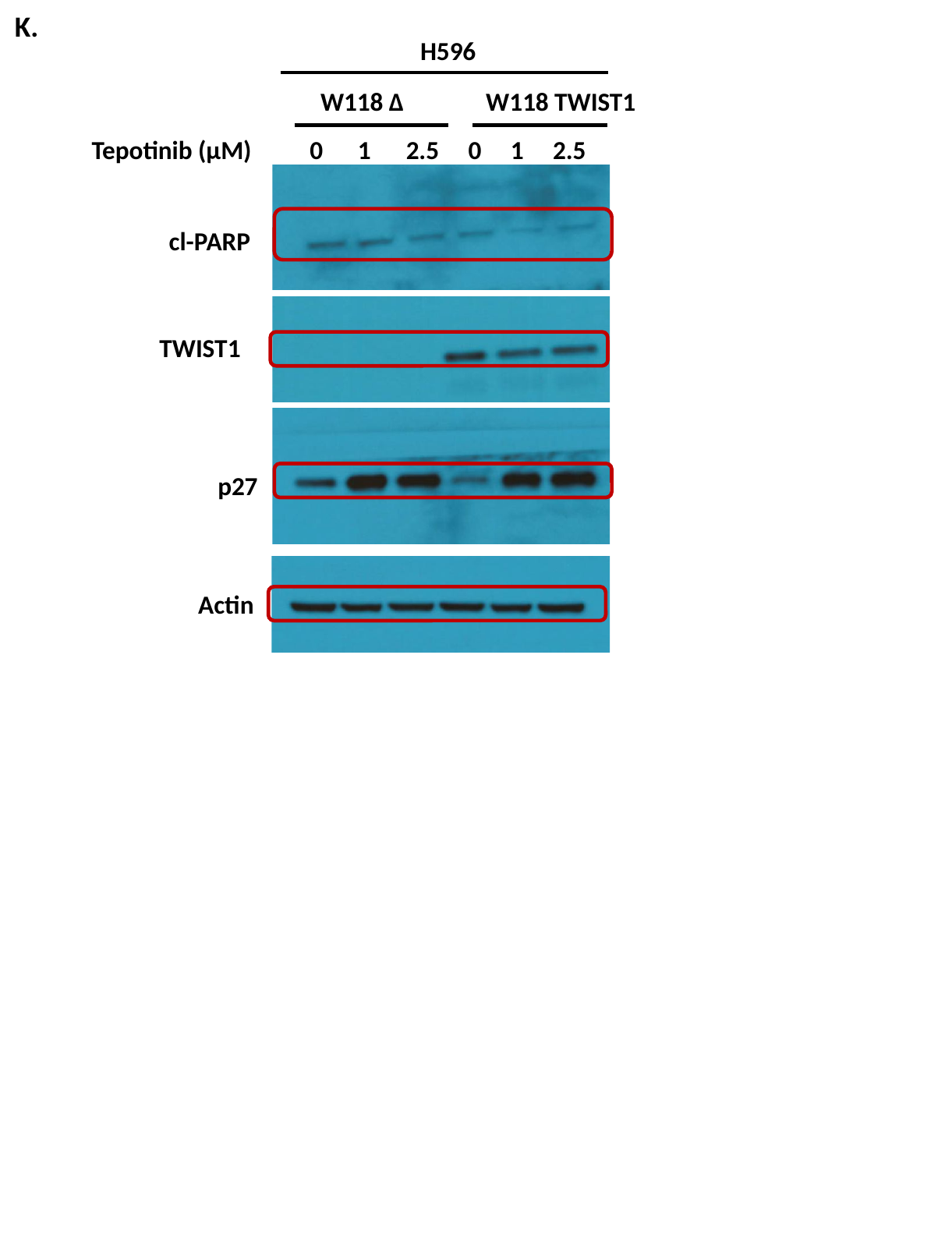

K.
H596
 W118 Δ W118 TWIST1
 Tepotinib (µM) 0 1 2.5 0 1 2.5
cl-PARP
TWIST1
p27
Actin

## Slide 8
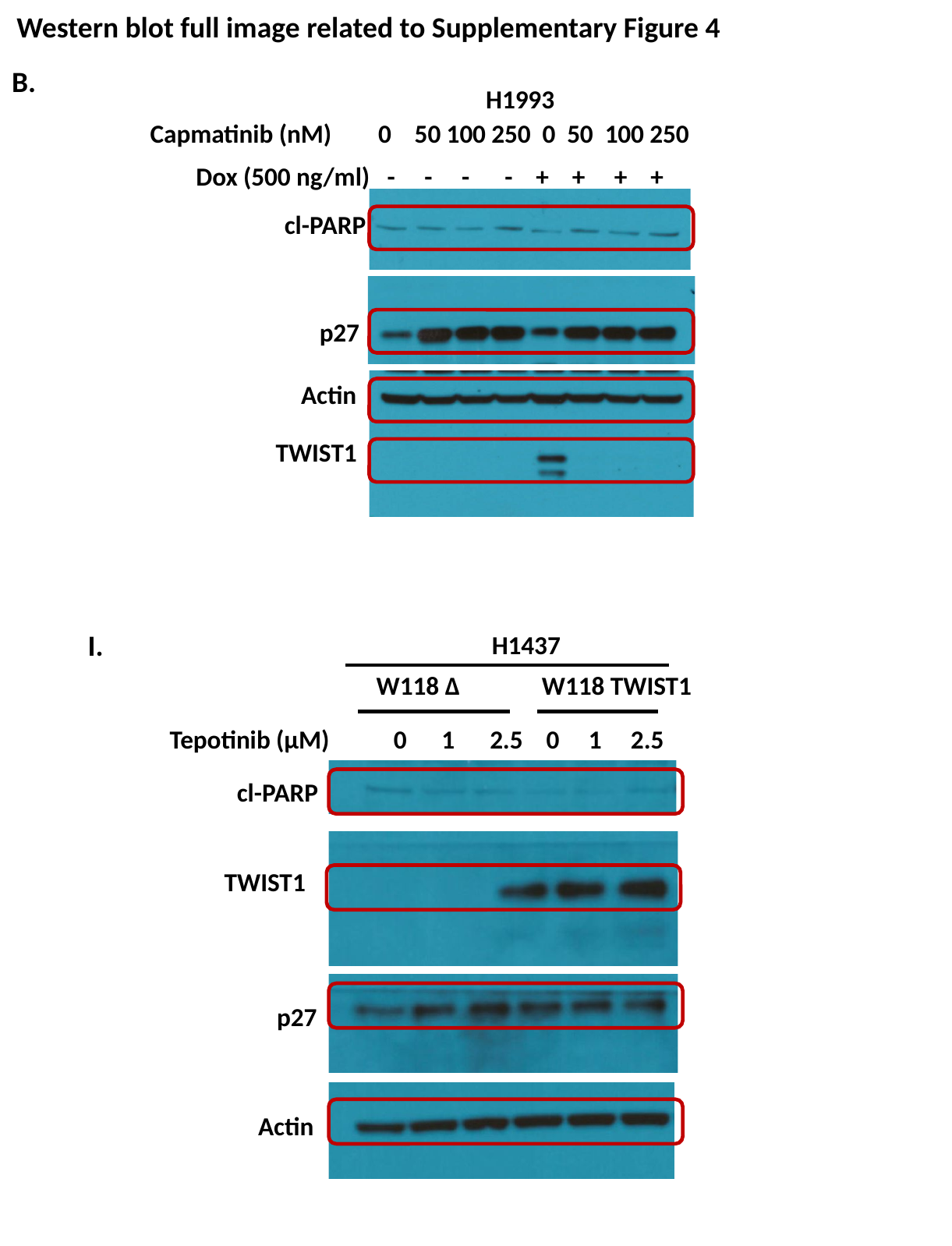

Western blot full image related to Supplementary Figure 4
B.
H1993
 Capmatinib (nM) 0 50 100 250 0 50 100 250
 Dox (500 ng/ml) - - - - + + + +
cl-PARP
p27
Actin
TWIST1
I.
H1437
 W118 Δ W118 TWIST1
 Tepotinib (µM) 0 1 2.5 0 1 2.5
cl-PARP
TWIST1
p27
Actin

## Slide 9
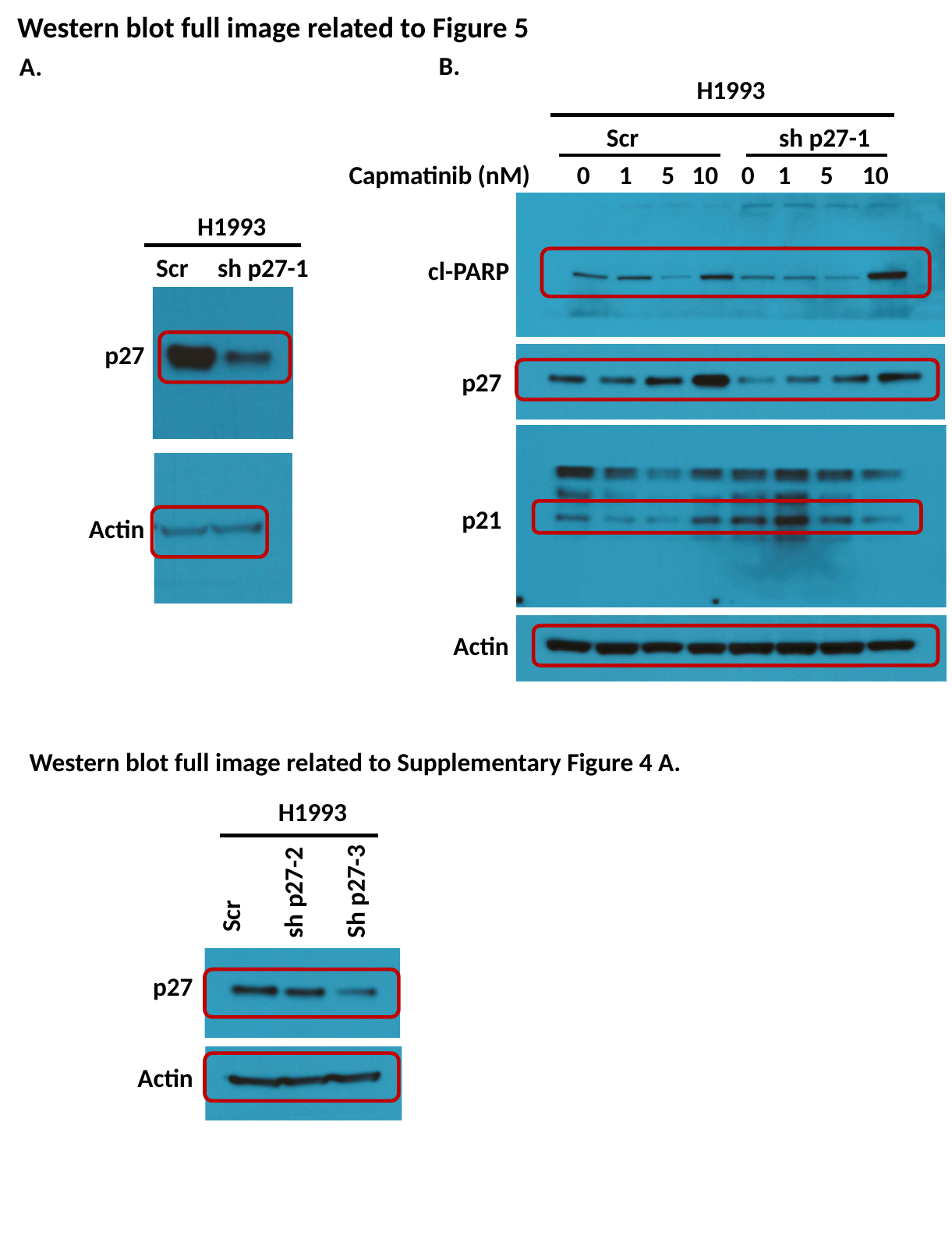

Western blot full image related to Figure 5
B.
A.
H1993
 Scr sh p27-1
 Capmatinib (nM) 0 1 5 10 0 1 5 10
cl-PARP
p27
p21
Actin
H1993
 Scr sh p27-1
p27
Actin
Western blot full image related to Supplementary Figure 4 A.
H1993
 Scr
sh p27-2
Sh p27-3
p27
Actin

## Slide 10
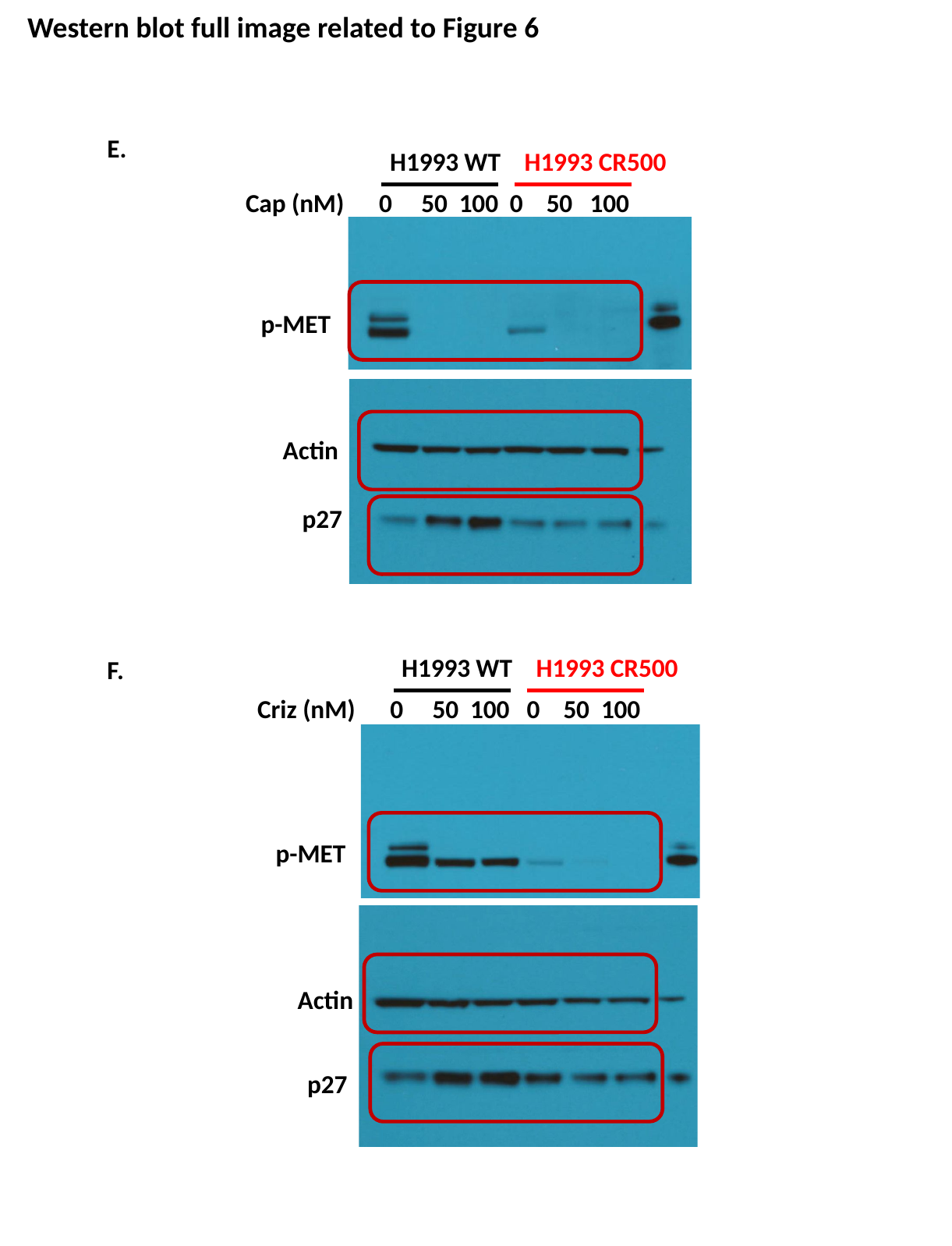

Western blot full image related to Figure 6
E.
 H1993 WT H1993 CR500
 Cap (nM) 0 50 100 0 50 100
p-MET
Actin
p27
 H1993 WT H1993 CR500
F.
 Criz (nM) 0 50 100 0 50 100
p-MET
Actin
p27

## Slide 11
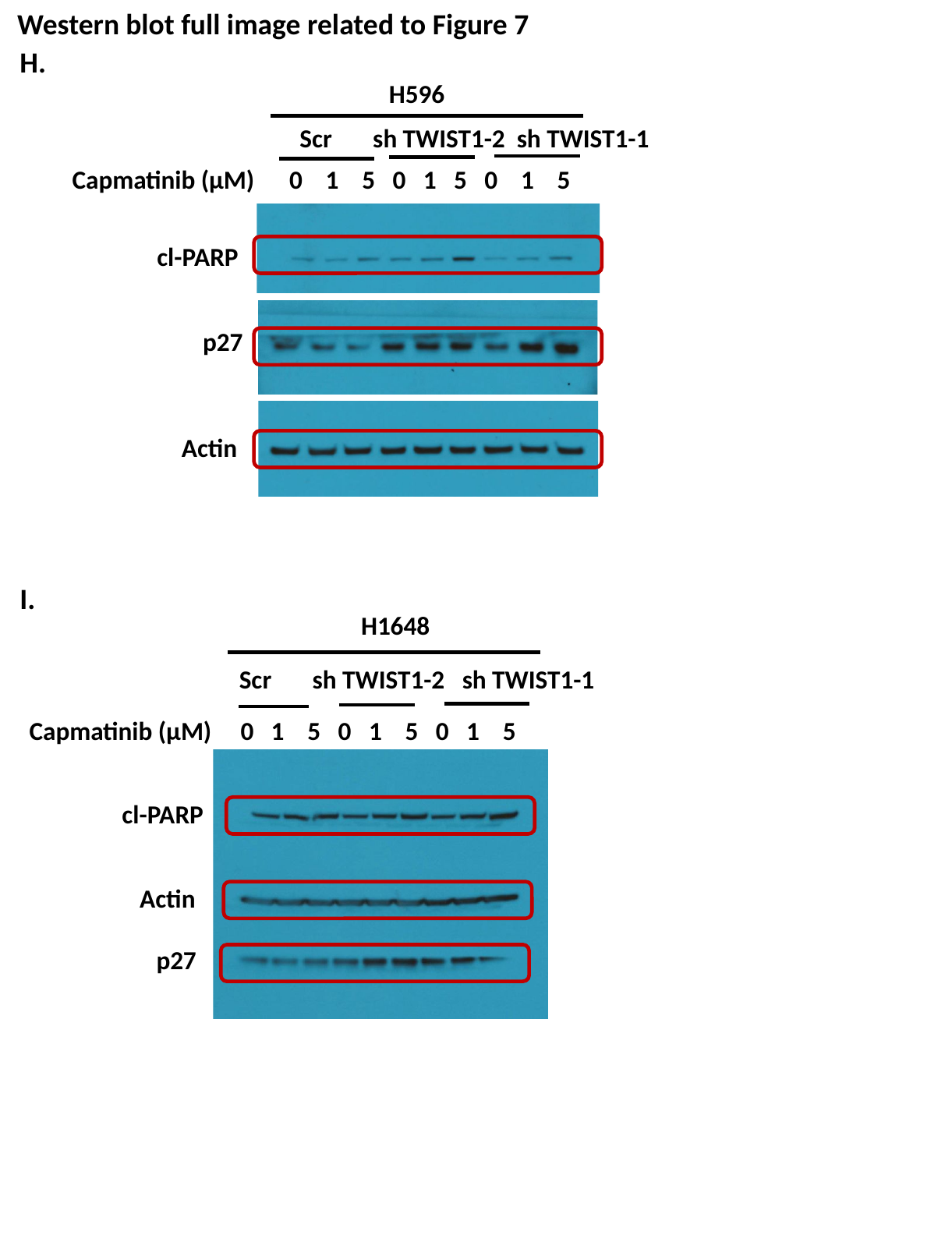

Western blot full image related to Figure 7
H.
H596
Scr sh TWIST1-2 sh TWIST1-1
Capmatinib (µM) 0 1 5 0 1 5 0 1 5
cl-PARP
p27
Actin
I.
H1648
 Scr sh TWIST1-2 sh TWIST1-1
Capmatinib (µM) 0 1 5 0 1 5 0 1 5
cl-PARP
Actin
p27

## Slide 12
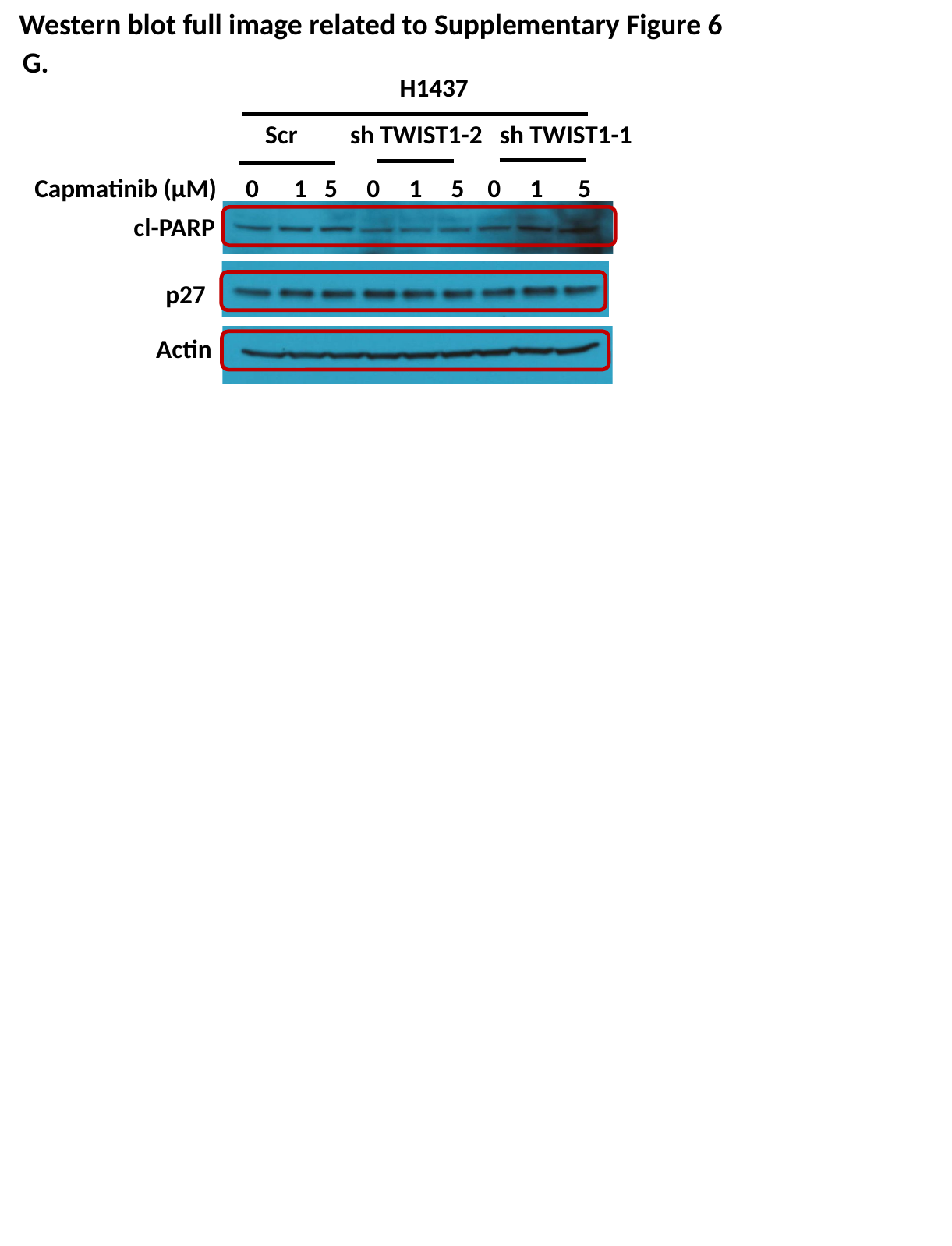

Western blot full image related to Supplementary Figure 6
G.
H1437
 Scr sh TWIST1-2 sh TWIST1-1
Capmatinib (µM) 0 1 5 0 1 5 0 1 5
cl-PARP
p27
Actin
